# RNA-Mediated Thermoregulation of Iron-Acquisition Genes in *Shigella dysenteriae* and Pathogenic *Escherichia coli*

**DOI:** 10.1371/journal.pone.0063781

**Published:** 2013-05-21

**Authors:** Andrew B. Kouse, Francesco Righetti, Jens Kortmann, Franz Narberhaus, Erin R. Murphy

**Affiliations:** 1 Ohio University, Department of Biology, Molecular Cell Biology Program. Athens, Ohio, United States of America; 2 Ruhr University Bochum, Microbial Biology. Bochum, Germany; 3 Ohio University Heritage College of Osteopathic Medicine, Department of Biomedical Sciences. Athens, Ohio, United States of America; University of Louisville, United States of America

## Abstract

The initiation, progression and transmission of most bacterial infections is dependent upon the ability of the invading pathogen to acquire iron from each of the varied environments encountered during the course of a natural infection. In total, 95% of iron within the human body is complexed within heme, making heme a potentially rich source of host-associated nutrient iron for invading bacteria. As heme is encountered only within the host, pathogenic bacteria often regulate synthesis of heme utilization factors such that production is maximal under host-associated environmental conditions. This study examines the regulated production of ShuA, an outer-membrane receptor required for the utilization of heme as a source of nutrient iron by *Shigella dysenteriae,* a pathogenic bacterium that causes severe diarrheal diseases in humans. Specifically, the impact of the distinct environmental temperatures encountered during infection within a host (37°C) and transmission between hosts (25°C) on *shuA* expression is investigated. We show that *shuA* expression is subject to temperature-dependent post-transcriptional regulation resulting in increased ShuA production at 37°C. The observed thermoregulation is mediated by nucleic acid sequences within the 5′ untranslated region. In addition, we have identified similar nucleotide sequences within the 5′ untranslated region of the orthologous *chuA* transcript of enteropathogenic *E. coli* and have demonstrated that it also functions to confer temperature-dependent post-transcriptional regulation. In both function and predicted structure, the regulatory element within the *shuA* and *chuA* 5′ untranslated regions closely resembles a FourU RNA thermometer, a zipper-like RNA structure that occludes the Shine-Dalgarno sequence at low temperatures. Increased production of ShuA and ChuA in response to the host body temperature allows for maximal production of these heme acquisition factors within the environment where *S. dysenteriae* and pathogenic *E. coli* strains would encounter heme, a host-specific iron source.

## Introduction

*Shigella* species are gram-negative, facultative intracellular bacteria and the causative agents of shigellosis, a severe diarrheal disease in humans. The World Health Organization conservatively estimates the global health burden of shigellosis to be 165 million infections and 1.1 million deaths annually [1], [2]. With an infectious dose estimated to be as small as ten bacteria, shigellosis is commonly spread directly from person-to-person via the fecal-oral route of infection, although intermediates such as contaminated food or water have been shown to transmit the infection [3]. Once ingested, *Shigella* transit the gastrointestinal system of the human host to the colon, where a shigellosis infection is initiated by invasion of the bacterium into cells within the colonic epithelium [2]. In addition to profuse bloody diarrhea and intense inflammation, the primary symptoms of infection, shigellosis is also associated with the development of hemolytic uraemic syndrome (HUS) and Reiter’s syndrome, causing sequelae to the kidneys, cardiovascular system and joints of the infected host [4], [5].

During infection within and transit between hosts, *Shigella* species, like most pathogenic bacteria, must acquire iron to survive. Binding of iron within molecules such as heme, hemoglobin, myoglobin, transferrin and ferritin, effectively reduce the concentration of bioavailable iron within the human host to levels that are too low to support the growth of most bacterial pathogens [6], [7]. In response, bacteria have evolved to produce specialized high affinity uptake systems to mediate the acquisition of nutrient iron from heme and other host-associated iron sources [7], [8]. *Shigella* species contain several conserved iron uptake systems [9], [10], [11], [12], [13], [14], [15], [16], [17], [18], [19], [20]; however, unique to *S. dysenteriae* is the Shu (Shigella Heme Uptake) system, a system dedicated to the utilization of heme and heme containing proteins as sources of nutrient iron [13], [14], [21], [22].

The *S. dysenteriae* Shu system is encoded within a single chromosomal locus predicted to contain two monocistronic (*shuA* and *shuS*) and two polycistronic (*shuTWXY* and *shuUV*) transcripts driven by four iron-regulated promoters [13]. The *shuA* gene encodes an outer-membrane heme receptor that binds and imports heme into the periplasm in a TonB dependent manner [14], [21]. Once inside the periplasm, the heme moiety is bound by ShuT and is transported to the cytoplasmic heme binding protein, ShuS, via the ABC transporter containing ShuU and ShuV in a coupled action [23]. Within the cytoplasm, it is proposed that ShuS either transports heme to a heme-degradation protein to utilize the molecule as a source of nutrient iron or to a heme-binding protein to prevent heme induced toxicity [24]. The functions of the remaining genes within the shu locus, *shuWXY* has not yet been deduced yet these genes are highly conserved across *S. dysenteriae* strains. Within pathogenic strains of *E. coli* the Chu system, an ortholog to the Shu system, has also been shown to mediate the utilization of iron from heme [25].

Given the abundance of heme within the human host (approximately 95% of the total iron [26]), and the importance of iron for survival, the ability of pathogenic bacteria to utilize heme as a source of nutrient iron is often correlated with virulence [27], [28], [29], [30], . While the impact of the Shu system on the ability of *S. dysenteriae* to cause disease in the human host has not been directly tested, several lines of evidence suggest this system promotes virulence. First, the entire *shu* locus is conserved between *S. dysenteriae* and several pathogenic strains of *Escherichia coli* but is absent in non-pathogenic *E. coli* K-12 [13], [29], [31]. Furthermore, the presence of the *shuA* ortholog *chuA* in uropathogenic, enterohemorrhagic and enteroaggregative *E. coli*, has been positively correlated with virulence by both *in vitro* analysis and by epidemiological studies of naturally occurring human infections [13], [29], [31], [34]. Finally, the pathophysiology of shigellosis, specifically the resulting physical damage and bleeding from the colonic epithelium, creates an environment in which *S. dysenteriae* will encounter heme. Together, these facts strongly suggest that the ability of *S. dysenteriae* to utilize heme as a source of nutrient iron provides the pathogen a competitive advantage within the human host.

While iron is essential for the survival of *S. dysenteriae*, and heme represents a potentially rich source of iron for the bacterium, both iron and heme are toxic at high concentrations [35]. The potential toxicity of iron and heme forces the bacterium to maintain a precise balance between the nutritional requirements for iron and the toxic effects of over-accumulation; an iron disequilibrium can lead to death of the bacterium [35]. Furthermore, it is energetically advantageous for the pathogen to produce a heme acquisition system only when it is within the host, as this is the sole environment in which the organism will encounter heme. For these reasons, the production of bacterial heme acquisition and utilization systems is often regulated in response to multiple host-associated environmental conditions including iron limitation, the presence of heme and/or host body temperature [25], [27], [30], [36], [37], [38], [39], [40], [41].

The influence of environmental conditions in controlling the expression of genes within the *shu* locus has focused primarily on the impact of iron availability on expression of *shuA,* the gene encoding an outer-membrane heme receptor [13], [14], [21]. Previous studies have demonstrated that *shuA* transcription is regulated in response to iron availability by the activity of the iron-dependent transcriptional repressor Fur, such that maximal expression is observed under iron-limited conditions [14], [21]. While these previous studies implicated iron-availability in modulating *shuA* expression, the impact of additional host-associated environmental conditions on *shuA* expression had not been investigated.

Several lines of evidence suggest that temperatures may influence *shuA* expression. First, temperature-dependent regulation of iron-acquisition systems has been observed in several other bacterial species [27], [40]. Second, temperature varies significantly between the heme-containing environment of the human-host (37°C) and the non-heme containing environment encountered during transmission from one host to the next (25°C). Lastly, the prediction of a promoter located over 300 bases from the translational start site suggests the presence of an extended 5′ untranslated region (utr) within the *shuA* mRNA molecule [13], [14]. An extended 5′ utr is a feature common among cis-acting RNA thermometers where RNA base pairing is known to regulate the expression of virulence and heat-shock genes in response to environmental temperature [42]. Such zipper-like RNA thermometers form inhibitory structures that function to occlude the ribosome binding site until the bacterium invades into a warm-blooded host or sudden heat shock causes melting of the inhibitory structure and permits translation initiation. [43], [44], [45], [46], [47], [48], [49], [50], [51], [52].

The goals of this study were to determine if the expression of *S. dysenteriae shuA,* and the *E. coli* ortholog *chuA,* is regulated in response to environmental temperature and to determine the molecular mechanism underlying any observed thermoregulation.

## Materials and Methods

### Bacterial Strains, Plasmids, Primers and Culture Conditions

All bacterial strains and plasmids used in this study are shown in Table 1. *E. coli* was routinely cultured in Luria-Bertani (LB) broth (1% tryptone 0.5% yeast extract and 1% NaCl) or on LB agar plates at 37°C. *S. dysenteriae* was routinely cultured in LB broth or on tryptic soy broth agar plates (Becton Dickenson and Company, Sparks, MD) containing 0.01% (wt/vol) Congo red dye (ISC BioExpress, Kaysville, UT) at the indicated temperature. Iron-limited growth conditions were achieved by the addition of 200 µg/ml of deferrated ethylenediamine-N,N’-bis 2-hydroxyphenylacetic acid (EDDHA) to LB. EDDHA was deferrated as detailed previously [53]. Chloramphenicol and Ampicillin were used at a final concentration of 30 µg/mL and 150 µg/mL respectively to select for the presence of reporter or control plasmids. All oligonucleotide primer sequences used in this study are available upon request.

**Table 1:** Bacterial Strains and Plasmids.

Designation	Description	Reference/Source
**Strains**		
***S. dysenteriae***		
ND100	Wild-type *S. dysenteriae.* Spontaneous Str^r^ mutant of clinical strain O-4576S1	[56]
Δ*shuA*	*shuA* deletion in O-4576S1	[14]
Δ*fur*	*fur* deletion in ND100	[56]
***E. coli***		
DH5α		Invitrogen
Top10		Invitrogen
**Plasmids**		
pXG-10	Low-copy plasmid containing a PLtetO-1 constitutive promoter driving expressionof a *gfp* reporter; Chl^r^	[54]
p*shuA*-*gfp*	pXG-10 lacking the constitutive promoter with the full length *shuA* 5′ utr and promotertranslationally fused to the *gfp* reporter; Chl^r^	This study
pWT-*shuA*	pXG-10 containing the *shuA* FourU element from *S. dysenteriae* translationallyfused to the *gfp* reporter; Chl^r^	This study
pS-*shuA*	pWT-shuA containing a stabilizing mutation within the FourU element; Chl^r^	This study
pD-*shuA*	pWT-shuA containing a destabilizing mutation within the FourU element; Chl^r^	This study
pBAD2-*bgaB*	Optimized version of pBAD-*bgaB* containing the pBAD promoter driving expressionof a *bgaB* reporter gene with a nonfunctional ATG; Amp^r^	[58]
p*agsA*	pBAD2-*bgaB* containing the *agsA* 5′-UTR from *Salmonella* translationally fusedto the *bgaB* reporter; Amp^r^	[58]
p*gyrA*	pBAD2-*bgaB* containing the *gyrA* 5′-UTR from *E. coli* translationally fused to the*bgaB* reporter; Amp^r^	This study
pWT-*chuA*	pBAD2-*bgaB* containing *chuA* 5′-UTR from *E. coli* UTI89 (UPEC) translationally fusedto the *bgaB* reporter; Amp^r^	This study
pS-*chuA*	pWT-*chuA* containing a stabilizing mutation within the FourU element; Amp^r^	This study
pD-*chuA*	pWT-*chuA* containing a destabilizing mutation within the FourU element; Amp^r^	This study

### Generation of the Gfp Translation Reporter Plasmid pshuA-gfp

Following isolation from *E. coli* strain Top10 using a Plasmid Midi Kit (Qiagen, Valencia, CA) according to manufacturer protocols, plasmid pXG-10 [54] was digested with AatII and NheI restriction endonucleases (New England BioLabs, Ipswich, MA) to remove the PLtetO-1 promoter and *lacZ* fragment. A DNA fragment containing the *shuA* start codon and 390 upstream nucleotides was amplified from the chromosome of wild-type *S. dysenteriae* by polymerase chain reaction using primers which contain AatII and NheI endonuclease recognition sites respectively. The amplified product was purified using a QIAQuick gel extraction kit (Qiagen) and then digested with AatII and NheI endonucleases and cloned into the digested pXG-10 plasmid backbone to create p*shuA*-*gfp*. The nucleic acid sequence of p*shuA*-*gfp* was verified by nucleic acid sequencing of both DNA strands.

### Generation of the Truncated Wild-type and Mutant Translational Reporter Plasmids pWT-shuA, pS-shuA and pD-shuA

After extraction of pXG-10 from *E. coli* strain Top10 with a Plasmid Midi Kit (Qiagen, Valencia, CA) according to manufacturer protocols, the plasmid was digested with NsiI and NheI to maintain the PLtetO-1 constitutive promoter and remove the *lacZ* fragment. DNA fragments encoding wild-type and mutant FourU RNA elements were created by the annealing of complementary DNA oligonucleotides generating an insert with nucleic acid sequences at the 5′ and 3′ ends resembling sites digested with NsiI and NheI endonucleases, respectively. Complementary oligonucleotides were boiled for 10 minutes in 1×STE buffer and allowed to cool to room temperatures. After annealing, the oligonucleotides were ligated into the pXG-10 backbone creating the translational fusion between the inserted sequence and the *gfp* reporter yielding plasmids pWT-*shuA*, pS-*shuA* and pD-*shuA*. Each plasmid generated was verified by nucleic acid sequencing of both strands to confirm insertion of the correct DNA fragments.

### Creation of β-galactosidase Reporters pWT-chuA, pS-chuA and pD-chuA

293 nucleotides 5′ of the *chuA* translational start site, containing the *chuA* 5′ utr and the first two codons of the gene were amplified from uropathogenic *E. coli* UT189 (UPED NC_007946.1) via PCR using primers which introduce NheI and EcoRI restriction sites into the amplified product. Plasmid pBAD2-*bgaB* and the amplified *chuA* 5′ utr were digested using NheI and EcoRI (Thermo Scientific, Fermentas, St. Leon-Rot, Germany) and ligated together forming the translational fusion and generating plasmid pWT-*chuA*. Mutations were introduced into the *chuA* sequence carried on plasmid pWT-*chuA* using the QuikChange® mutagenesis kit (Stratagene, La Jolla, USA) according to manufacturer protocols. Specifically, within pWT-*chuA* the thymine at position −19, relative to the *bgaB* translational start site, was mutated to a cytosine to create pS-*chuA*, and an adenine to create pD-*chuA*. Plasmids were sequence verified by Eurofins (Eurofins, Martinsried, Germany).

### β-galactosidase Assays

*E. coli* DH5α cells carrying the *bgaB* reporter plasmids pWT-*chuA,* pS-*chuA* and pD-*chuA* were grown overnight in 5 mL Luria-Bertani (LB) medium supplemented with ampicillin (150 µg/mL) at 25°C. 1 ml of the stationary phase culture was used to inoculate a 25 mL LB culture which was grown to an OD_600_ of 0.5 prior to the addition of 0.01% arabinose (w/v) to induce transcription from each reporter plasmid. Next, 10 mL of each culture was shifted to pre-warmed flasks at 37°C and 400 µL of sample taken after 30 minutes of incubation. β-Galactosidase assays were performed on each collected sample as described by Rinnenthal et al. [55]. Standard deviations were calculated from three independent experiments.

### Western Blot Analysis

All Western blot analyses were performed using whole cell extracts. Bacterial cultures were grown to stationary phase under the indicated growth conditions and the optical density at 600 nm measured using a ND-1000 spectrophotometer (NanoDrop Technologies, Wilmington, DE). A total of 5×10^8^ bacterial cells from each culture were pelleted and suspended in 200 µL of Laemmli protein dye (Bio-Rad) containing 5% 2-mercaptoethanol and were boiled for 10 minutes. Samples were stored frozen at −20°C until use.

ShuA Western blot analysis was carried out as follows: 15 µL of each whole cell protein preparation was separated by sodium dodecyl sulfate polyacrylamide gel electrophoresis (SDS-PAGE) using a 7.5% polyacrylamide gel and was then transferred to a PVDF membrane that had been pre-soaked in methanol for 10 minutes and rinsed three times with water. Following protein transfer, the membrane was blocked by incubation overnight at 4°C in a solution of phosphate buffered saline with 0.1% Tween20 (PBST) and 5% milk. After blocking, the membrane was washed 4 times for 5 minutes each time using PBST and then incubated for 1 hour at 4°C with polyclonal α-ShuA IgG (Custom antibody raised in a rabbit - Fisher Scientific, Waltham, MA) diluted 1:1000 in a solution of PBST with 5% milk. Following a series of washes as described above, the membrane was incubated for 1 hour at 4°C with goat anti-rabbit HRP conjugated IgG (Bio-Rad) diluted 1:10,000 in a solution of PBST with 5% milk. Finally, the membrane was rinsed as above, incubated in Immun-Star regents (Bio-Rad) according to manufacturer protocols and imaged using x-ray film.

Gfp Western blot analysis was carried out as detailed for that of ShuA with the following modifications. Following transfer of whole-cell protein samples to the PVDF, as detailed above, the membrane was blocked by incubation overnight at 4°C in a solution of PBST and 10% milk. Next, the membrane was incubated for one hour at 4°C with anti-Gfp monoclonal IgG stabilized antibody preparation (Roche, Indianapolis, IN) diluted 1:1000 in a solution of PBST and 5% milk. The membrane was then washed 3 times for 5 minutes, incubated in a solution of PBST and 10% milk for 10 minutes at 4°C and then incubated for 1 hour at 4°C with goat anti-mouse HRP conjugated IgG (Bio-Rad) diluted 1:20,000 in PBST with 5% milk. Finally, the membrane was washed and the blot was imaged using Immun-Star WesternC reagents (Bio-Rad) and a ChemiDoc XRS+ Imaging System (Bio-Rad). Contrast was altered uniformly over the entire image using Image Lab software (Bio-Rad) to reduce background noise and did not alter relative band intensity or the banding pattern observed.

### RNA Extraction

Following growth of the bacterial culture to the indicated growth phase under the specified growth condition and at the indicated temperature, total RNA was extracted using an RNEasy® Mini-kit (Qiagen) according to manufacturer protocols. The purified RNA was then treated with 16 units of amplification grade Dnase I (New England BioLabs) for 1 hour at 37°C. One mL of 100% EtOH, 40 µl of 3 M sodium acetate (pH 5.2) and 100 µL of 1 mM ethylenediaminetetraacetic acid (EDTA) were added to each sample prior to incubation overnight at −80°C. Following precipitation of the RNA by centrifugation at 12,000×g for 15 minutes at 4°C, each sample was washed with cold 75% EtOH, the RNA was pelleted as above and dried. The RNA pellet was resuspended in diethyl polycarbonate (DEPC) treated water and the nucleic acid concentration measured using an ND-1000 spectrophotometer (NanoDrop Technologies). Finally, to ensure the removal of contaminating DNA, each RNA sample was treated using the TURBO DNA-*free* kit (Ambion, Austin, TX) according to the manufacturer protocols. The lack of DNA in each RNA sample was confirmed by PCR using the purified RNA as template and oligonucleotide primers known to amplify a portion of the *shuA* open reading frame.

### Quantitative Real-Time Polymerase Chain Reaction

cDNA was generated from 150 ng of total RNA using the iScript cDNA Synthesis Kit (Bio-Rad) according to manufacturer protocols. Each cDNA sample was diluted 1:10 in water and 5 µL used as template in a 20 µL amplification reaction. Primer concentration and reaction conditions were optimized for each primer set. All quantitative Real-time PCR reactions were carried out using iQ SYBR Green Supermix (Bio-Rad). A six point standard curve was generated for each target during each experimental run to ensure that an acceptable efficiency was achieved in the analysis. All expression values were calculated using the ΔΔCt method, normalized to the level of *rrsA* measured in each sample and expressed relative to the value obtained in the indicated control sample. All reactions were performed in a Bio-Rad CFX96 Real-Time PCR System. All primer sequences were designed using Beacon Designer 7.5 and are available upon request.

### Reverse Transcriptase PCR

A two-step reverse transcriptase PCR was conducted in a PTC-200 DNA Engine Cycler (Bio-Rad) using SuperScript III Reverse Transcriptase (Invitrogen, Grand Island, New York) according to manufacturer protocols followed by an amplification reaction. Using 150 ng of RNA isolated from wild-type *S. dysenteriae* cultured to stationary phase under iron-limited conditions as template, *shuA* specific cDNA was generated in the reverse transcription step using primer shu2 that binds within the *shuA* open reading frame. In the amplification step, specific products were amplified in a 50 µL reaction containing 1 µL template (cDNA), 5 µL of 2.5 mM dNTP, 5 µL of 10× Standard Taq Reaction Buffer, 1 µL of Taq DNA Polymerase, 36 µl of RNase free water, 1 µL of primer shu2 and 1 µL of either primer shu3, primer shu4 or primer shu6 at a final concentration of 0.6 µM of each primer. A “No-RT control” in which the isolated RNA was used as template in the amplification step was performed with each primer pair to screen for DNA contamination in the RNA sample. A positive amplification control using primer pair shu2 and shu6 was conducted using *S. dysenteriae* genomic DNA as template. Amplification products were resolved on a 2% agarose gel with 0.001% ethidium bromide and visualized with a ChemiDoc XRS+ Imaging System (Bio-Rad).

### Alignment and in silico Modeling

Nucleic acid sequences for alignment of the promoters and 5′ untranslated regions of *shuA* and *chuA* were acquired from GenBank. Alignments were conducting using Clone Manager 9 software and were recreated in Adobe Illustrator CS4. Predicted structure of *shuA* 5′ utr, partial and complete, were obtained using Mfold software (http://mfold.rna.albany.edu/?q=mfold/RNA-Folding-Form).

### Statistical Analysis

Two tailed, two sample Student’s t tests, assuming equal variance, were used throughout our studies to determine significance (P≤0.05).

## Results

### ShuA Production is Influenced by Environmental Temperature

**Figure 1:**
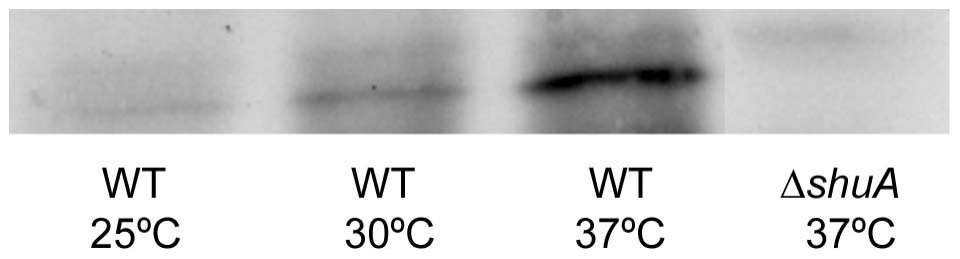
ShuA protein production is influenced by environmental temperature with increased levels detected at 37°C. Wild-type *S. dysenteriae* and *S. dysenteriae* Δ*shuA* knockout strain were grown to stationary phase at the temperatures indicated under iron limiting conditions (LB media containing 200 µg/mL EDDHA). Western blot analyses were conducted using whole-cell lysates generated from an equivalent number of bacteria grown under each condition and a polyclonal anti-*shuA* antibody (custom generated, Fisher Scientific).

The impact of environmental temperature on ShuA levels was assessed directly by Western blot analysis using a polyclonal anti-ShuA antibody. ShuA levels were measured in whole cell extracts prepared from an equal number of wild-type *Shigella dysenteriae* (ND100 [56]) cells following growth to stationary phase under iron-limited conditions at 25°C, 30°C and 37°C. Iron-limited growth conditions were used for this study to relieve Fur-mediated repression of *shuA* transcription [14]. The data demonstrate a positive correlation between environmental temperature and ShuA levels with increased ShuA levels present in *S. dysenteriae* following growth of the strain at 37°C as compared to those measured following growth of the strain at either of the lower temperatures tested (Figure 1). A strain of *S. dysenteriae* lacking *shuA* (Δ*shuA*) was used as a negative control in this assay and as expected, shows no specific reactivity with the anti-ShuA antibody (Figure 1). These data indicate that ShuA levels are modulated in response to environmental temperature with increased ShuA levels achieved at the host-associated temperature of 37°C as compared to the non-host associated temperatures of 25°C and 30°C.

### Temperature-dependent Modulation of ShuA Production is Mediated by a Post-transcriptional Regulatory Mechanism

**Figure 2:**
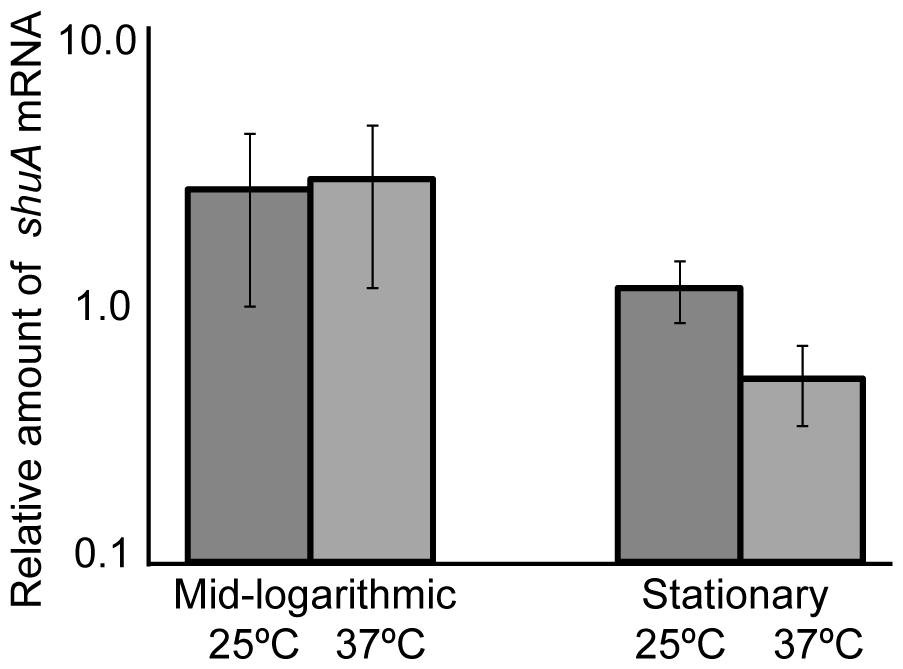
*shuA* mRNA levels are not significantly altered by changes in environmental temperature. Quantitative Real-time PCR was conducted using RNA extracted from wild-type *S. dysenteriae* cultured to the mid-logarithmic or stationary phases of growth in LB broth containing 200 µg/mL EDDHA at 25°C and 37°C. *shuA* mRNA levels were normalized to the amount of *rrsA*, a constitutively expressed gene, in each sample and expressed relative to the amount of *shuA* transcript measured in the first 25°C sample at stationary phase. These data are the average of three biological replicates and error bars represent one standard deviation. Assuming a confidence interval of 95% (p≤0.05), no significant difference exists between the relative amounts of *shuA* mRNA measured at 25°C and 37°C.

In order to determine which step within the *shuA* expression pathway is thermoregulated, the effect of environmental temperature on the relative abundance of *shuA* mRNA molecules was investigated by Quantitative Real-Time PCR (Q-PCR) analysis. *shuA* transcript levels were measured following growth of wild-type *S. dysenteriae* to mid-logarithmic or stationary phase at 25°C and 37°C under iron limiting conditions. The temperatures of 37°C and 25°C were chosen for this analysis because they represent the physiologically relevant temperatures encountered by the bacterium during an infection and during transmission, respectively, and because it is at these two temperatures that the greatest difference in ShuA protein levels was observed (Figure 1). Unlike ShuA protein levels, *shuA* transcript levels are not significantly higher following growth of wild-type *S. dysenteriae* at 37°C as compared to that following growth of the strain at 25°C (Figure 2). These data indicate temperature does not significantly influence the rate of *shuA* transcription or the stability of the *shuA* transcript. Temperature-dependent modulation of ShuA protein levels in the absence of parallel alteration in *shuA* mRNA levels indicates that the observed thermoregulation is mediated by a post-transcriptional regulatory mechanism.

### Nucleic Acid Sequences within the shuA 5′ Untranslated Region (utr) Confer Thermo-regulation of ShuA Translation

**Figure 3:**
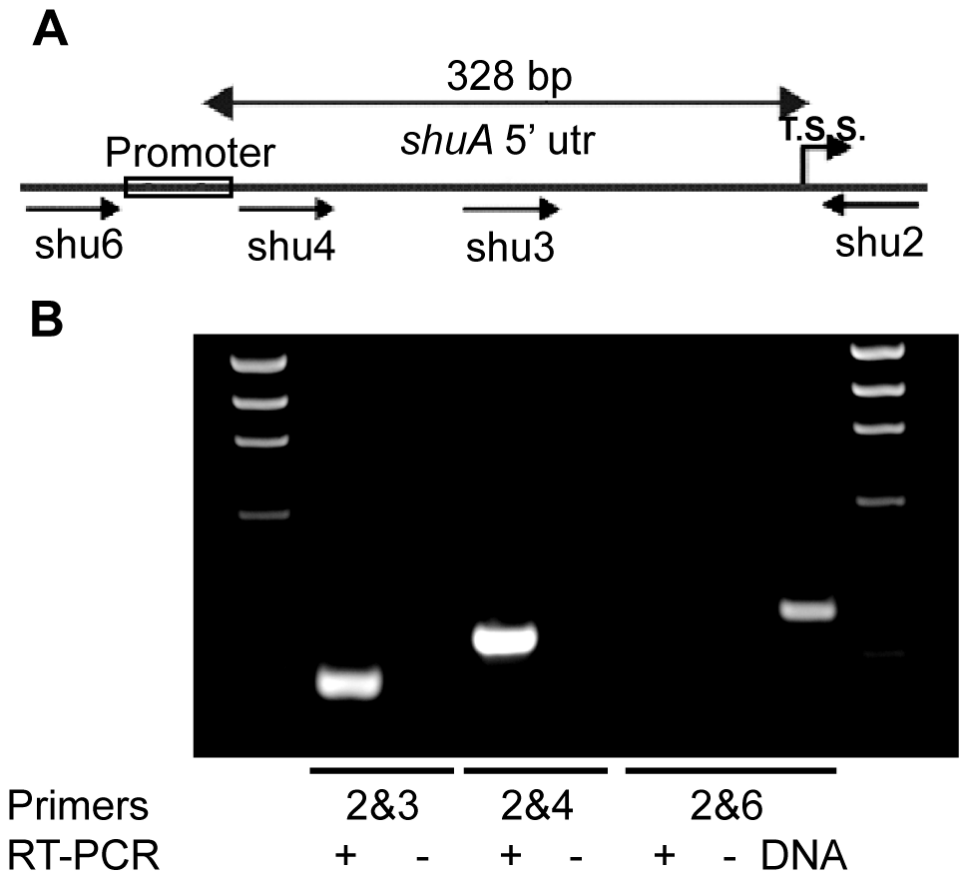
The *shuA* transcript contains an extended 5′ untranslated region. **A)** **** Schematic (not drawn to scale) of the *S. dysenteriae shuA* promoter and predicted 328-nucleotide 5′ utr. Arrows represent primer-binding sites used in the cDNA synthesis and amplification steps of the reverse transcription polymerase chain reaction (RT-PCR) analyses. The predicted promoter region is boxed and the translational start site (T.S.S.) is indicated. **B)** The predicted *shuA* 5′ utr was experimentally confirmed by a series of RT-PCR analyses using RNA isolated from wild-type *S. dysenteriae* grown under iron limiting conditions (LB media containing 200 µg/mL EDDHA). For each experimental reaction, primer shu2 was used to generate the *shuA* specific cDNA product. In each amplification step, the reverse primer shu2 was paired with the indicated forward primer. A control reaction using RNA as template was conducted with each primer set to ensure that the isolated RNA sample did not contain DNA contamination; a (−) indicates RNA was used as template in the amplification reaction while a (+) indicates that cDNA was used as template in the amplification reaction. DNA was used as template in the amplification step to verify that the indicated primer pair was capable of facilitating target amplification.

An alteration of translational efficiency is often controlled by nucleotides surrounding or composing the Shine-Dalgarno (SD) sequence within the target mRNA (often within a 5′ utr) [44], [47], [48], [49]. To determine the influence of nucleotides within the *shuA* 5′ utr on the observed temperature-dependent modulation of ShuA levels, the existence of the previously predicted *shuA* 5′ utr and its ability to confer thermoregulation was investigated. The existence of the predicted *shuA* 5′ utr was confirmed by reverse transcriptase PCR (RT-PCR) analysis (Figure 3). These data demonstrate that the *shuA* 5′ utr is between 278 and 342 nucleotides in length, a length that is in good agreement with the 328 nucleotide 5′ utr previously predicted by sequence analysis [13].

**Figure 4:**
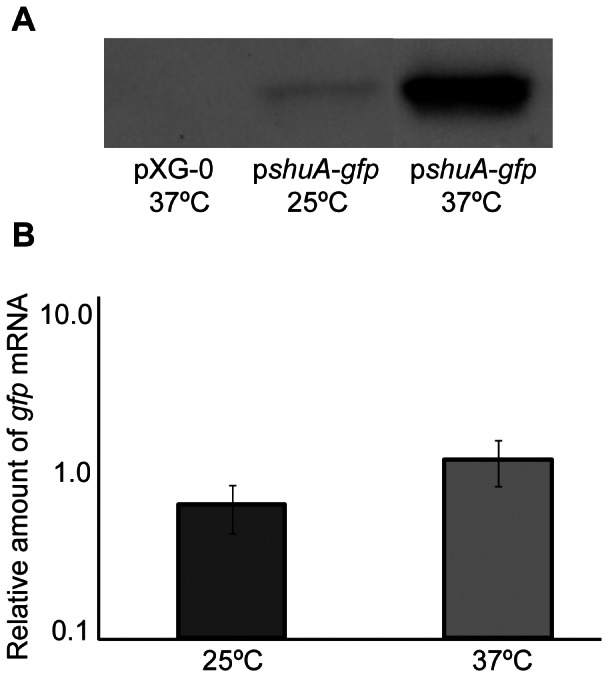
The *shuA* promoter and 5′ utr are sufficient to confer temperature-dependent post-transcriptional regulation. **A)** **** A Western blot analysis was performed with monoclonal anti-Gfp antibodies and whole-cell extracts generated from an equivalent number of wild-type *S. dysenteriae* carrying either p*shuA*-*gfp* or the empty vector pXG-0. All strains were cultured under iron-limited conditions (LB with 200 µg/mL EDDHA) to stationary phase at the temperatures indicated. **B)** Quantitative Real-time PCR was carried out using RNA isolated from wild-type *S. dysenteriae* carrying p*shuA*-*gfp* cultured at the indicated temperature under iron-limited conditions (LB with 200 µg/mL EDDHA). *gfp* mRNA levels were normalized to the amount of *rrsA* measured in each sample and expressed relative to the amount of *gfp* transcript in the first 25°C sample. All data are representative of three biological replicates and error bars represent one standard deviation. Assuming a confidence interval of 95% (p≤0.05), no significant difference exists between the relative levels of *gfp* transcript measured from p*shuA*-*gfp* following growth of the strain at 25°C or 37°C.

Next, nucleic acid sequences composing the full-length 5′ utr and *shuA* promoter were cloned into a modified pXG-10 [54] reporter plasmid to generate p*shuA*-*gfp*, a plasmid carrying a translational fusion between the first *shuA* codon and a functional *gfp* reporter gene. From this plasmid, expression of *gfp* is controlled by the native *shuA* promoter and may be influenced by nucleic acid sequences within the *shuA* 5′ utr. The effect of temperature on *gfp* expression from p*shuA*-*gfp* was investigated by a combined approach using both Western blot and Q-PCR analyses following growth of wild-type *S. dysenteriae* carrying p*shuA*-*gfp* under iron limiting conditions to stationary phase at 25°C and 37°C. Western blot analysis using whole cell extracts generated from equivalent numbers of wild-type *S. dysenteriae* carrying p*shuA*-*gfp* demonstrated that Gfp protein levels are higher following growth of the strain at 37°C as compared to those seen following growth of the strain at 25°C (Figure 4A). Q-PCR analysis demonstrated that temperature does not significantly alter the relative abundance of *gfp* mRNA present in wild-type *S. dysenteriae* carrying the p*shuA*-*gfp* reporter plasmid (Figure 4B). The observed temperature-dependent alteration of Gfp protein levels in the absence of a corresponding change in *gfp* transcript levels from the p*shuA*-*gfp* translational reporter plasmid indicate that nucleic acid sequences within the *shuA* promoter and 5′ utr are sufficient to confer post-transcriptional temperature-dependent regulation onto the expression of the *gfp* reporter gene.

### In silico Analysis Predicts the Presence of a FourU RNA Thermometer within the shuA 5′ utr

**Figure 5:**
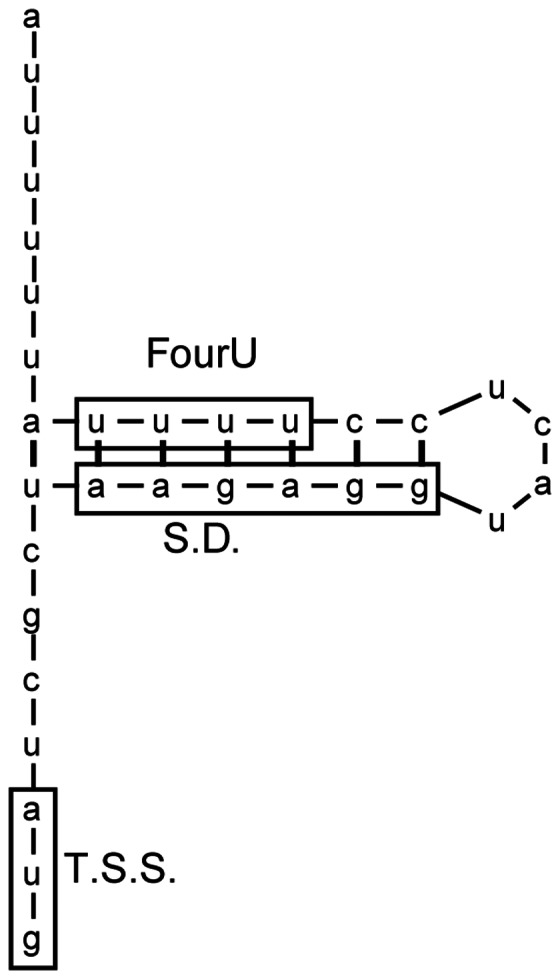
*In-silico* folding analysis predicts a FourU thermometer occluding the Shine-Dalgarno sequence within the *shuA* mRNA. Nucleic acid sequence and secondary structure of nucleotides −29 through +3 of *shuA* as predicted by Mfold analysis (http://mfold.rna.albany.edu). Boxes indicate the location of the four consecutive uracil residues and putative Shine-Dalgarno sequence within the FourU RNA thermometer, as well as the translational start site (T.S.S.) of *shuA*.

Given that nucleic acid sequences composing the *shuA* promoter and 5′ utr are sufficient to confer post-transcriptional thermoregulation, (Figure 4) the predicted secondary structure of this region was evaluated by *in silico* modeling to identify putative regulatory elements. MFold analysis was used to predict the secondary structure of the entire *shuA* 5′ untranslated region (http://mfold.rna.albany.edu/?q=mfold/RNA-Folding-Form), the most energetically favorable of which is shown in Figure S1. Ten out of twelve structures generated by Mfold included a short hairpin that is formed by canonical and non-canonical base-pairing between four consecutive uracil residues and the predicted Shine-Dalgarno sequence (Figure 5 and Figure S1). These characteristics are found in FourU RNA thermometers, a relatively new class of cis-acting riboregulators shown to mediate post-transcriptional thermoregulation of target gene expression by occlusion of the ribosomal binding site at non-permissive temperatures [42], [45], [55], [57].

### The Nucleotide Sequence Composing the Putative shuA FourU RNA Thermometer is Sufficient to Confer Post-transcriptional Thermoregulation

**Figure 6:**
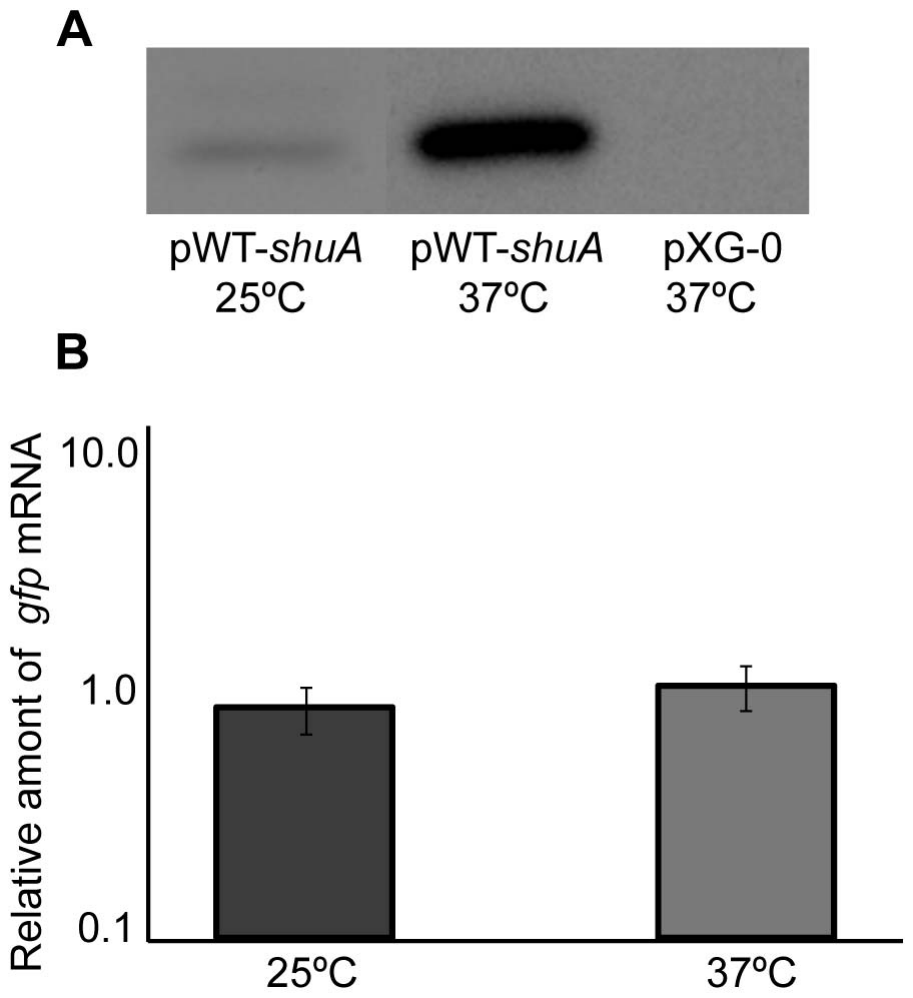
Sequences composing the *shuA* FourU thermometer are sufficient to confer post-transcriptional thermoregulation onto *gfp* expression. **A)** **** *E. coli* strain DH5α containing pWT-*shuA* or pXG-0, as a vector control, was cultured to stationary phase at the temperatures indicated. A Western blot was performed using whole-cell lysates generated from an equal number of cells and anti-Gfp monoclonal antibodies. **B)** Quantitative Real-time PCR was conducted using RNA isolated from *E. coli* DH5α carrying pWT-*shuA* following growth of the strain to stationary phase at 25°C or 37°C. *gfp* mRNA levels were normalized to *rrsA* measured in each sample and expressed relative to the amount of *gfp* transcript measured in the first 25°C sample. All data are representative of three biological replicates and error bars represent one standard deviation. Assuming a confidence interval of 95% (p≤0.05), no significant difference exists between the relative levels of *gfp* transcript measured from pWT-*shuA* following growth of the strain at 25°C or 37°C.

To determine if the nucleotide sequence comprising the predicted RNA thermometer is sufficient to confer post-transcriptional thermoregulation onto reporter gene expression, a 32 nucleotide long insert containing the putative FourU RNA thermometer (Figure 5) was cloned between the constitutive PLtetO-1 promoter and the *gfp* reporter gene on plasmid pXG-10. Such cloning generates a translational fusion between the first codon of *shuA* and *gfp*, the transcription of which is driven by the plasmid promoter and protein production is potentially influenced by the putative *shuA* FourU thermometer cloned into pXG-10. The newly created plasmid, designated pWT-*shuA*, was introduced into *Escherichia coli*, and thermoregulation of *gfp* expression was investigated by Western blot and Q-PCR analyses following growth of the strain to stationary phase at 25°C or 37°C (Figure 6). Western blot analyses demonstrate that Gfp protein levels are increased following growth of *E. coli* carrying pWT-*shuA* to stationary phase at 37°C as compared to those measured following growth of the strain at 25°C (Figure 6A) while Q-PCR analyses indicate that temperature does not influence the relative abundance of *gfp* mRNA (Figure 6B). Together, these data clearly demonstrate that the predicted FourU RNA thermometer contained within the *shuA* 5′ utr is sufficient to confer post-transcriptional thermoregulation onto the production of the Gfp reporter protein.

### Site-directed Mutagenesis of the Putative FourU Element Alters Thermoregulation of the gfp Reporter Gene

**Figure 7:**
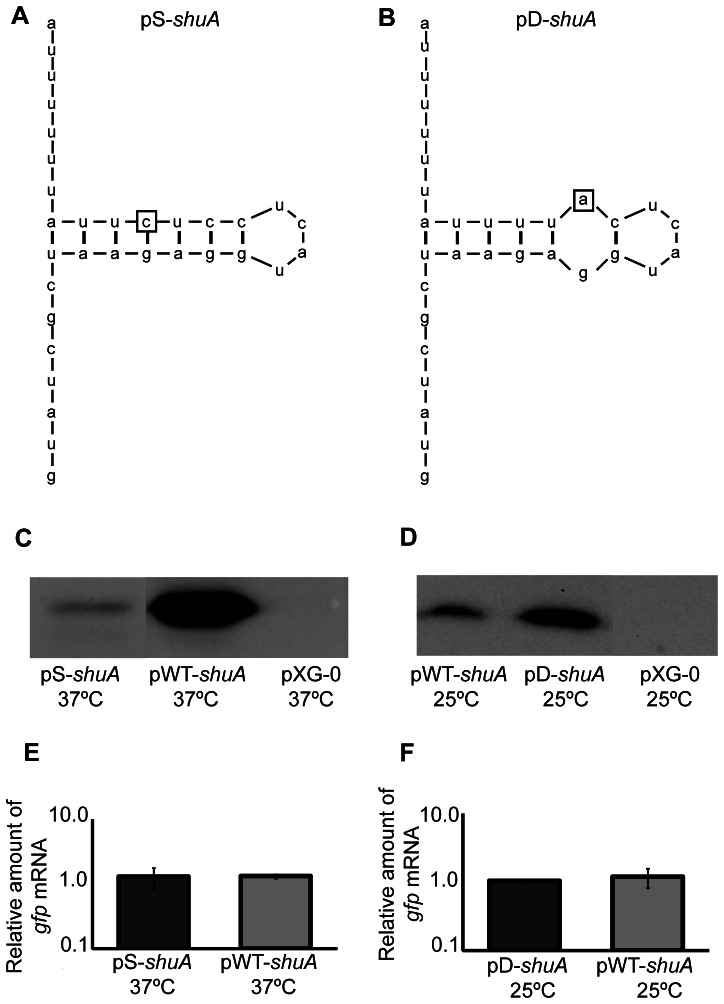
Mutational analysis demonstrates that the *shuA* 5′ utr contains a functional FourU RNA thermometer. The *shuA* region cloned into pWT-*shuA* was mutagenized to further validate the existence of a predicted FourU element within the *shuA* 5‘utr. **A)** The uracil residue located 19 nucleotides upstream of the *gfp* translational start site was mutated to a cytosine. This mutation, indicated by the box, is predicted to stabilize the inhibitory structure within the putative FourU RNA thermometer. This mutated sequence was cloned into the *gfp* translational reporter pXG-10 to generate the stabilized mutant construct designated pS-*shuA*. **B)** The cytosine residue 17 nucleotides upstream of the *gfp* translational start site was mutated to an adenine. This mutation, indicated by the box, is predicted to destabilize the inhibitory structure within the putative FourU RNA thermometer. This mutated sequence was cloned into the *gfp* translational reporter pXG-10 to generate the destabilized mutant construct designated pD-*shuA*. Western blot analyses were conducted using monoclonal anti-Gfp antibodies and whole-cell extracts generated from an equal number of *E. coli* carrying pWT-*shuA* or pS-*shuA* cultured to stationary phase in LB at the permissive temperature of 37°C (**C**), and *E. coli* carrying pWT-*shuA* or pD-*shuA* cultured to stationary phase in LB at the non-permissive temperature of 25°C (**D**). Quantitative real-time PCR analysis was performed using RNA isolated from *E. coli* DH5α cells carrying pWT- *shuA,* pS-*shuA* and pD*-shuA* after culturing the strains to stationary phase using the temperatures indicated. *gfp* transcript levels were normalized to the amount of *rrsA* in each sample and set relative to the amount of *gfp* in the first pWT-*shuA* sample. All data are representative of three biological replicates and error bars represent one standard deviation. Assuming a confidence interval of 95% (p≤0.05), no significant difference exists between the relative levels of *gfp* transcript measured from pWT-*shuA* and pS-*shuA* (**E**) or pD-*shuA* (**F**) at the temperatures tested.

To further verify the existence of a FourU RNA thermometer within the *shuA* 5′ utr, single nucleotide mutations were introduced into the regulatory element cloned within the pWT-*shuA* reporter plasmid. The first mutant construct, pS-*shuA*, contains a thymine to cytosine mutation to generate a cytosine-guanine base pair within the predicted RNA thermometer (Figure 7A). This mutation was created to stabilize the closed conformation of the FourU RNA thermometer, a mutation that is predicted to decrease reporter protein production from pS-*shuA* compared to pWT-*shuA* when measured at the permissive temperature of 37°C. *E. coli* was transformed with pS-*shuA* and Gfp production compared to that from pWT-*shuA* by Western blot analysis following the growth of each strain at 37°C. Gfp production from pS-*shuA* following growth of the reporter strain at the permissive temperature of 37°C is inhibited as compared to that from the strain carrying pWT-*shuA* (Figure 7C). Q-PCR analysis demonstrates that *gfp* transcript levels produced from pS-*shuA* and pWT-*shuA* are equivalent under the conditions tested, indicating that the observed difference in Gfp levels does not result from altered transcription or transcript stability of the mutated reporter gene (Figure 7E). Together these data demonstrate that, as expected, mutations that are predicted to stabilize the inhibitory structure within the putative FourU RNA thermometer result in decreased expression of the regulated gene at the permissive temperature of 37°C.

The second mutant construct, pD-*shuA,* contains a cytosine to adenine mutation which is predicted to abolish a canonical cytosine-guanine base-pair and destabilize the inhibitory structure of the FourU RNA thermometer thus allowing for increased Gfp production at the non-permissive temperature of 25°C (Figure 7B). *E. coli* was transformed with pD-*shuA* and Gfp production compared to that from pWT-*shuA* by Western blot analysis following the growth of each strain at 25°C. Gfp production from pD-*shuA* following growth of the reporter strain at the non-permissive temperature of 25°C is increased as compared to that from the strain carrying pWT-*shuA* (Figure 7D). Q-PCR analysis demonstrates that *gfp* transcript levels produced from pD-*shuA* and pWT-*shuA* are equivalent under the conditions tested, indicating that the observed difference in Gfp levels does not result from altered transcription or transcript stability of the mutated reporter gene (Figure 7F). Together these data demonstrate that, as expected, mutations that are predicted to destabilize the inhibitory structure within the putative FourU RNA thermometer result in increased expression of the regulated gene at the non-permissive temperature of 25°C.

Collectively, these mutagenesis studies demonstrate that thermoregulation conferred by the putative *shuA* FourU RNA thermometer is mediated by differential stability of the identified inhibitory structure. These data strongly support the conclusion that the *shuA* 5′ utr harbors a functional FourU RNA thermometer.

### The 5′ utr of chuA, the E. coli Ortholog of shuA, Contains a FourU RNA Thermometer Sufficient to Confer Post-transcriptional Thermoregulation

**Figure 8:**
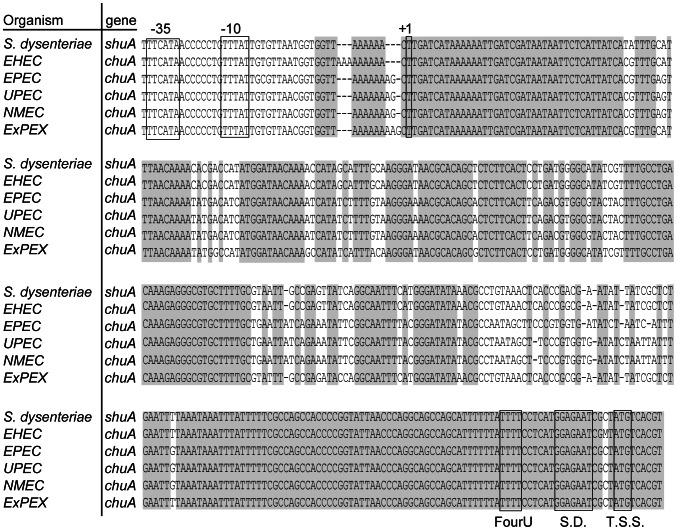
Nucleic acid sequences within the *shuA* 5′ utr are conserved among pathogenic *E. coli* species. An alignment of the *shuA* and *chuA* promoter sequence, 5′ utr and first nine nucleotides of the coding region are pictured. Nucleotides that are conserved throughout the *shuA* and *chuA* genes are highlighted. The predicted −35, −10, transcriptional start site (+1), FourU thermometer (FourU), Shine-Dalgarno (S.D.) sequence and translational start site (T.S.S.) are indicated by boxes. The alignment was performed using Clone Manager 9. An ‘m’ is an ambiguous nucleotide and denotes either a thymine or cytosine. All sequences were acquired from GenBank and are as follows: *shuA* from Shigella dysenteriae Sd197 NC_007606.1, *chuA* from Enterohemorrhagic E. coli (EHEC) NC_002655.2, *chuA* from Enteropathogenic E. coli (EPEC) NC_011601.1, *chuA* from Uropathogenic E. coli (UPEC) NC_007946.1, *chuA* from Neonatal Meningitis E. coli (NMEC) NC_011742.1, and *chuA* from Extraintestinal Pathogenic E. coli (ExPEC) NC_011751.1.

**Figure 9:**
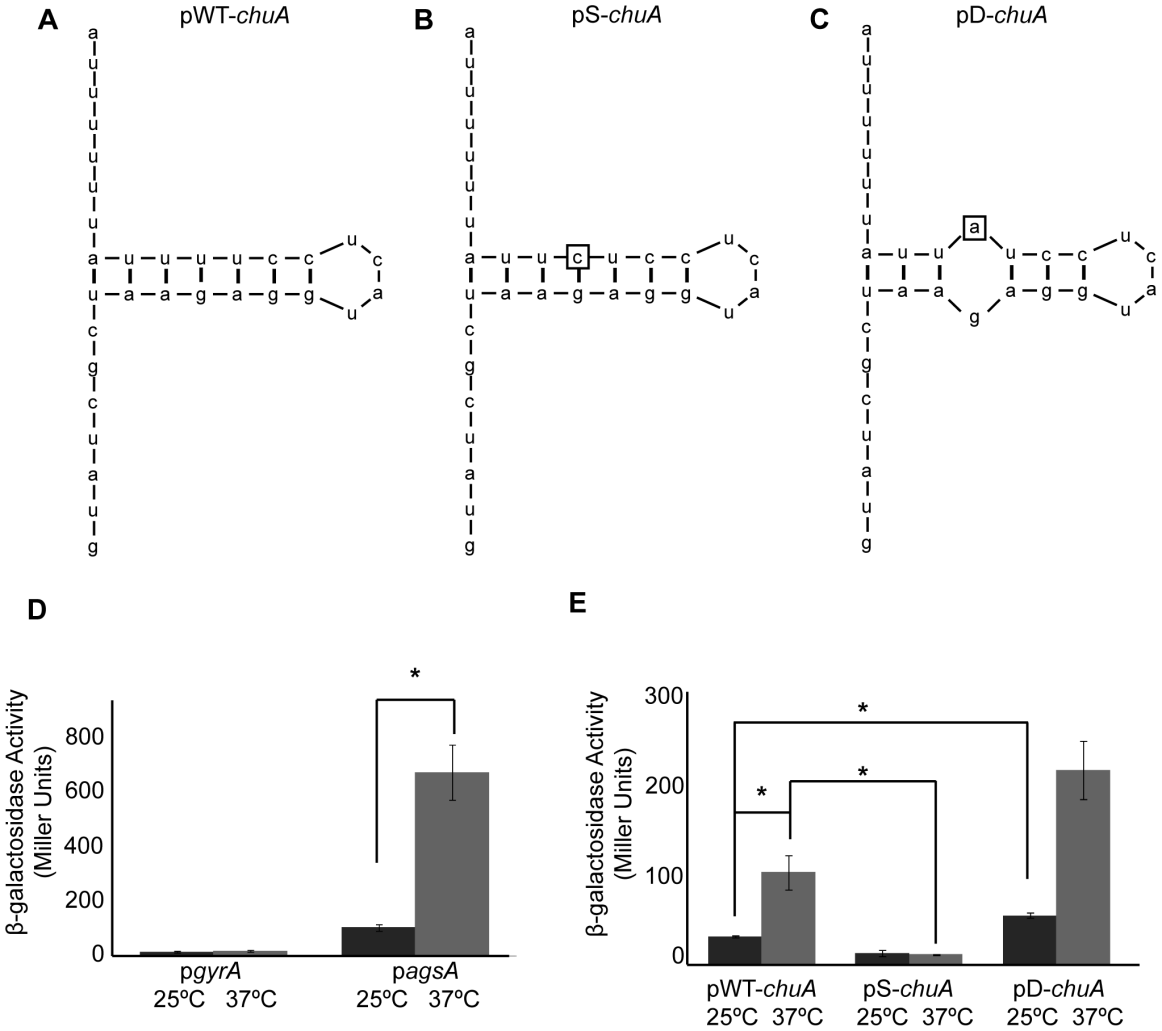
A functional FourU RNA thermometer within the 5′ utr of *E. coli* chuA confers thermoregulation. **A)** **** Predicted structure of a portion of the *chuA* mRNA molecule containing the start codon (AUG) and preceding 29 nucleotides. This region contains the putative *chuA* FourU RNA thermometer. **B)** The uracil residue located 19 nucleotides upstream of the *bgaB* translational start site was mutated to a cytosine. This mutation, indicated by the box, is predicted to stabilize the inhibitory structure within the putative *chuA* FourU RNA thermometer. The mutated sequence was cloned into the pBAD2-*bgaB* translational reporter to generate the stabilized mutant construct designated pS-*chuA*. **C)** The uracil residue located 19 nucleotides upstream of the *bgaB* translational start site was mutated to an adenine. This mutation, indicated by the box, is predicted to destabilize the inhibitory structure within the putative *chuA* FourU RNA thermometer. The mutated sequence was cloned into the pBAD2-*bgaB* translational reporter to generate the destabilized mutant construct designated pD-*chuA*. The influence of temperature on the expression of *bgaB* from the positive and negative control plasmids p*agsA* and p*gyrA* **D)** and the translational reporters pWT-*chuA,* pS-*chuA* and pD-*chuA* **E)** was determined by measuring the β-galactosidase activity produced form *E. coli* DH5α carrying each. All date are the average of three replicate experiments. Error bars represent one standard deviation and a *indicates a statistically relevant difference between the indicated strains (p≤0.05).

An alignment among the 5′ utr of *shuA* and its ortholog *chuA* from several strains of pathogenic *E. coli* shows moderate sequence variation; however, the FourU RNA thermometer and surrounding regions are completely conserved (Figure 8). To determine if the *E. coli chuA* 5′ utr is capable of conferring thermoregulation, this region was amplified from uropathogenic *E. coli* UT189 (UPEC NC_007946.1) and cloned into plasmid pBAD2-*bgaB*, a well-established translational reporter system [58]. Specifically, a region containing 293 nucleotides of the *chuA* 5′ utr and the first two codons of the open reading frame was cloned in frame with the *bgaB* reporter, encoding a heat-stable *β*-galactosidase, to generate the translational reporter plasmid pWT-*chuA.* Expression of *bgaB* from pWT-*chuA* is under control of the pBAD arabinose inducible promoter and is potentially influenced by the putative *chuA* FourU RNA thermometer contained within the cloned *E. coli* sequence (Figure 9A).

*E. coli* DH5α was transformed with pWT-*chuA* and the thermoregulation of *bgaB* activity investigated. A temperature shift from 25°C to 37°C resulted in a significant increase in the β-galactosidase activity measured from pWT-*chuA* (Figure 9E). These data demonstrate that like that of *S. dysenteriae shuA* (Figure 6), sequences within the 5′ utr of UPEC *chuA* are sufficient to confer thermoregulation onto the expression of a reporter gene on a translational reporter plasmid. Two control plasmids were utilized in this study. The negative control plasmid, p*gyrA*, carries the reporter *bgaB* translationally fused to the 5′ utr of the non-thermoregulated *E. coli gyrA* and is a modification of that used previously [45]. The positive control plasmid, p*agsA* [58], carries the reporter *bgaB* translationally fused to a previously characterized FourU RNA thermometer from *Salmonella agsA*. As expected, a temperature-dependent increase in β-galactosidase activity was also seen from the positive control plasmid p*agsA*, while no thermoregulation was observed from the negative control plasmid p*gyrA* (Figure 9D).

To further characterize the UPEC *chuA* FourU RNA thermometer, point mutations were introduced to stabilize or destabilize the predicted inhibitory structure cloned within the pWT-*chuA* reporter plasmid. Non-pathogenic *E. coli* DH5α was transformed with each mutated reporter plasmid and the influence of the mutation of thermoregulation of the reporter *bgaB* gene evaluated by β-galactosidase analyses. The first mutant construct, pS-*chuA*, carries a thymine to cytosine mutation that introduces a C-G pairing within the predicted RNA thermometer (Figure 9B). As predicted, this stabilizing mutation results in significantly less β-galactosidase activity from the pS-*chuA* translational reporter at the permissive temperature of 37°C as compared to that measured from pWT-*chuA* (Figure 9E). The second mutant construct, pD-*chuA*, carries a thymine to adenine mutation that destabilizes the predicted *chuA* FourU RNA thermometer by disrupting a U-A pairing (Figure 9C). This destabilizing mutation results in significantly more β-galactosidase activity from the pD-*chuA* translational reporter at the non-permissive temperature of 25°C as compared to that measured from pWT-*chuA* (Figure 9E). Together, these data indicate that, like that of *S. dysenteriae shuA,* the expression of UPEC *chuA* is regulated in response to environmental temperature by the activity of a FourU RNA thermometer located within the 5′ utr of the gene.

## Discussion

This study demonstrates that the expression of *S. dysenteriae shuA* and *E. coli chuA,* genes essential for the utilization of heme as a source of nutrient iron, is modulated in response to environmental temperature by a post-transcriptional regulatory mechanism. This work is the first to demonstrate temperature-dependent post-transcriptional regulation of *shuA* and *chuA* expression and the first to implicate temperature as an environmental factor controlling the production of an iron uptake system in *Shigella.* Finally, it is demonstrated that the observed post-transcriptional thermoregulation of both *shuA* and *chuA* expression is mediated by the activity of a conserved FourU RNA thermometer within the 5′ utr of each gene.

**Figure 10:**
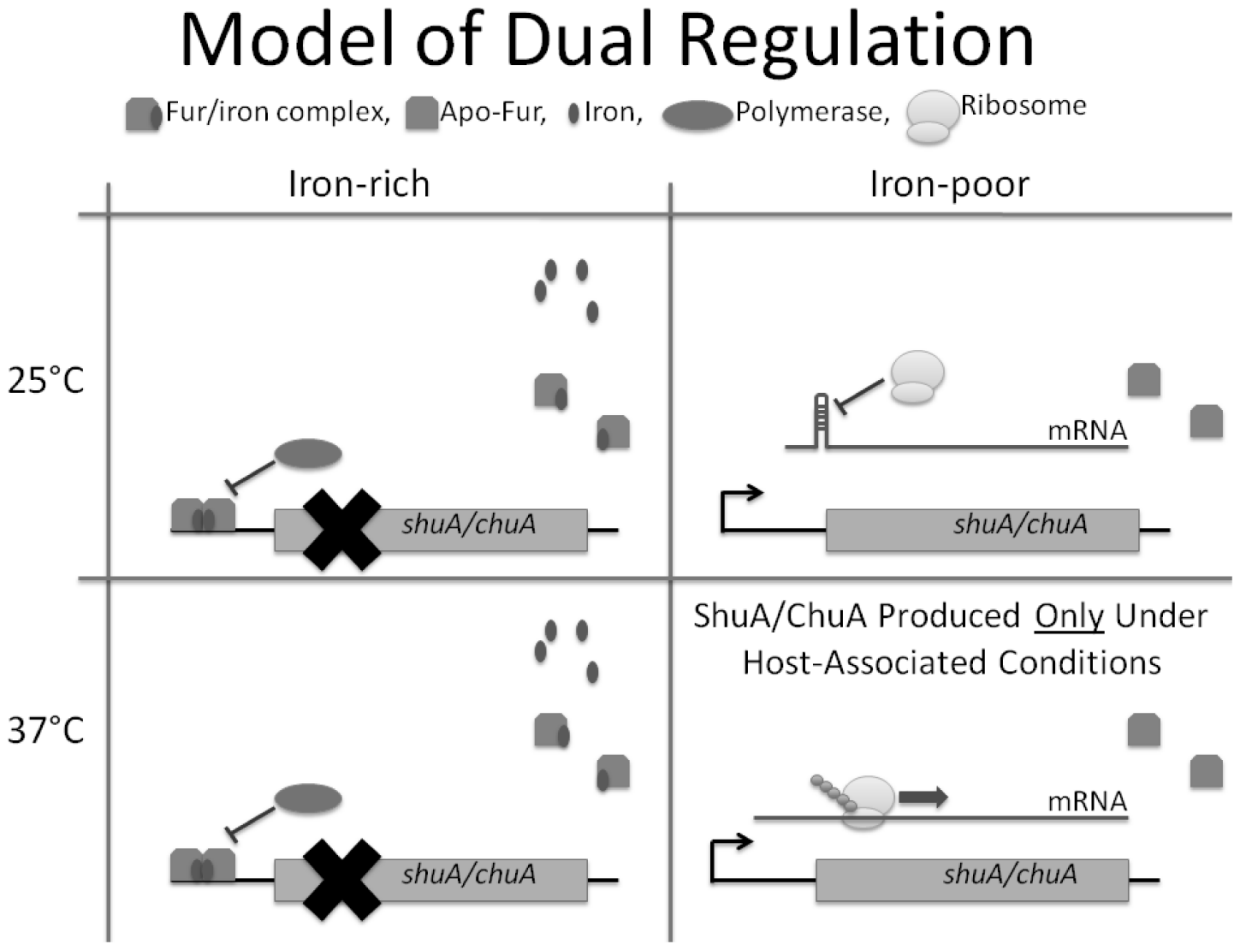
Dual regulation controlling the expression of *S. dysenteriae* *shuA* and *chuA* of pathogenic *E. coli*. The production of *S. dysenteriae* ShuA and *E. coli* ChuA is regulated at the levels of transcription and translation by two independent mechanisms. Transcription of the *shuA* and *chuA* message is inhibited under high-iron conditions by the iron-dependent transcriptional repressor Fur in a temperature-independent manner. However, under iron-poor conditions Fur mediated repression of *shuA/chuA* transcription is relieved and mRNA is synthesized. Translation of the *shuA/chuA* transcript is regulated in response to environmental temperature via a FourU RNA thermometer within the 5′ untranslated region of each gene. At a temperature of 25°C, translation of *shuA/chuA* is blocked by the formation of an inhibitory structure predicted to occlude the ribosomal binding site and prevents ribosomal binding. At a temperature of 37°C the inhibitory structure within the *shuA* and *chuA* 5′ utr is destabilized and efficient translation of each gene proceeds. This model shows that production of ShuA and ChuA occurs only under iron-poor conditions and at 37°C, conditions which mimic the host environment where *S. dysenteriae* and pathogenic *E. coli* will encounter heme as a rich source of nutrient iron.

The conservation of the FourU RNA thermometer between related pathogenic bacterial species (Figure 8) suggests that it provides a selective advantage by increasing the fitness of each species. Multiple regulatory mechanisms control the expression of both *S. dysenteriae shuA* and *E. coli chuA* and this hierarchical regulation likely increases the fitness of each pathogen by limiting the production of the heme binding receptor to environmental conditions mimicking those encountered within the heme containing human body (Figure 10). This work along with previous studies has demonstrated that the expression of *shuA* and *chuA* is regulated by both iron availability and environmental temperature [14], [25]. Dual-regulation of *shuA* and *chuA* likely increases the overall fitness of *S. dysenteriae* and *E. coli* by promoting expression of each gene within the host, where heme is going to be encountered, while efficiently preventing expression of each gene in the non-host environment where heme will not be encountered. Specifically, the iron-limitation and environmental temperature (37°C) encountered within the human-host relieves both the Fur-mediated inhibition of *shuA* transcription and FourU RNA thermometer mediated inhibition of *shuA* translation, respectively (Figure 10). Consequently, host-associated conditions are optimal for *shuA* and *chuA* expression and subsequent heme acquisition by *S. dysenteriae* and pathogenic *E. coli*. Conversely, the non-host environment encountered during transmission, marked by an increase in available iron and a decrease in temperature, will result in Fur-dependent inhibition of *shuA* and *chuA* transcription and FourU RNA thermometer dependent inhibition of translation, respectively (Figure 10). As a result of this dual regulation, *shuA* expression will be efficiently inhibited during transmission through non-heme containing environments.

A further advantage that is likely conferred by the regulation of *shuA* and *chuA* expression at both the transcriptional and translational level of gene expression is that such regulation likely facilitates rapid adaptation of the pathogen to changing environmental conditions. It is reasonable to assume that upon being shed from an infected human host, *S. dysenteriae* and pathogenic *E. coli* experiences an increase in relative iron-availability and a decrease in environmental temperature. Inhibition of both transcription and translation would likely provide an advantage over regulation only at the level of transcription in this scenario, by preventing the translation of existing *shuA* and *chuA* mRNA molecules, thus saving the pathogen the energetic cost of producing the heme receptor in an environment in which the protein will provide no advantage to the bacterium.

While *shuA* is regulated at both the transcriptional and translational level of expression, additional regulatory mechanisms could influence *shuA* expression. The FourU element is located within the last 30 nucleotides of the fairly well conserved *shuA* 5′ utr that measures over 300 nucleotides (Figure 3). *In silico* folding analyses predicts a highly structured 5′ utr (Figure S1) that may play additional roles in *shuA* regulation. The impact of the remaining 5′ utr on *shuA* and *chuA* expression remains under investigation. It is plausible that additional elements within the 5′ utr of *chuA* and *shuA* are capable of stabilizing the FourU element and/or regulating expression of each gene in a manner independent of the FourU RNA thermometer.

Only two other FourU thermometers, *agsA* from *Salmonella* and *icrF* from *Yersinia* species, have been extensively characterized to date [45], [55], [57], [59]. Given the conservation and distribution of the *shu* locus and identified FourU RNA thermometer among *S. dysenteriae* and several strains of pathogenic *E. coli* (Figure S1) [13], as well as the predicted distribution of FourU RNA thermometers among both Gram-negative and Gram-positive bacterial species [45], the significance of this study reaches beyond *S. dysenteriae* gene regulation and into the broader fields of enteric pathogenesis and RNA-mediated mechanism of bacterial gene regulation.

Only after multiple FourU RNA thermometers are identified and characterized will a complete understanding of this potentially wide-spread regulatory element be possible. Future studies aimed at characterizing the regulatory function of the *shuA* FourU RNA thermometer in finer molecular detail as well as the identification and characterization of additional FourU RNA thermometers in *Shigella* and other bacterial species will not only further the understanding of this newly recognized regulatory element functions but will also reveal the potentially expansive role that FourU RNA thermometers play in bacterial physiology and pathogenesis.

## Supporting Information

Figure S1 ***In-silico*** **folding analysis of the full-length** ***shuA*** **5′ utr.** The full-length 5′ utr and start codon of *shuA* was submitted to Mfold for modeling of the RNA secondary structure. The predicted FourU RNA thermometer is denoted by a box.
(TIF)
