# Correction draft: RNA-Mediated Thermoregulation of Iron-Acquisition Genes in *Shigella dysenteriae* and Pathogenic *Escherichia coli*

**DOI:** 10.1371/journal.pone.0252744

**Published:** 2021-06-01

**Authors:** Andrew B. Kouse, Francesco Righetti, Jens Kortmann, Franz Narberhaus, Erin R. Murphy

After this article [1] was published, concerns were raised about the reported western blot results. Specifically:

- There appear to be vertical discontinuities between lanes in the following western blot panels: Figs 1 (after lane 3), 4A (after lane 2), 7C (after lane 1) and 7D (after lane 2). For each figure panel, the discontinuity reflects where nonadjacent image fragments from the same blot and exposure were spliced together in preparing the figure. Figs 1, 4 and 7 below have been updated to clarify that the panels represent spliced blots, and the original blot images are provided in S1–S4 Files.
- There appears to be a vertical discontinuity in Fig 6A (after lane 1) and the pWT-*shu*A 25°C band in Fig 6A appears similar to the pS-*shu*A 37°C band in Fig 7C, when rotated 180 degrees. Also, the background texture looks different in lane 1 versus in lanes 2–3 in Fig 6A. The underlying data confirm that errors were made during the construction of Fig 6A. These errors have been corrected in the updated Fig 6 below and the original blot has been provided in the S5 File. The consulted editor advised that the data provided for the corrected figure support the published results.
- The western blot experiments reported in the article did not include control blots to demonstrate relative loading across lanes. For each experiment, an equivalent amount of protein was loaded in each lane and equivalent loading was confirmed by staining the membrane with Ponceau S to visualize total protein. The Ponceau S staining data of the published panels are no longer available, instead repeat experiment data that include loading controls for Figs 4A, 6A, 7C and 7D are provided in the S7–S10 Files below. Fig 1 experiments could not be repeated anew due to degradation of the anti-ShuA antibody, but additional data from an older replicate showing similar results are provided in the S6 File.

An Academic Editor reviewed the updated figure and data files as well as the repeat experiment data and advised that the western blot results with the anti-GFP antibody demonstrate that *gfp* expression from the *shuA* promoter depends on temperature and the RNA hairpin structures. Furthermore, the adviser stated that the accompanying Ponceau S stainings of the membranes presented in the S7–S10 Files confirm equivalent protein loading. Regarding Fig 1, the Academic Editor noted that the overall protein levels appear to change significantly depending on growth temperature. Therefore, it is difficult to confirm the temperature-dependent expression of *shuA* based on the results presented in Fig 1, but the adviser confirmed that the results presented in Fig 4A support the temperature-dependent expression of *shuA*.

Original data underlying other results in this article may no longer be available due to the time that has passed.

The authors apologize for not indicating the image splicing lines in the published figures, and for the errors in Fig 6A.

**Fig 1:**
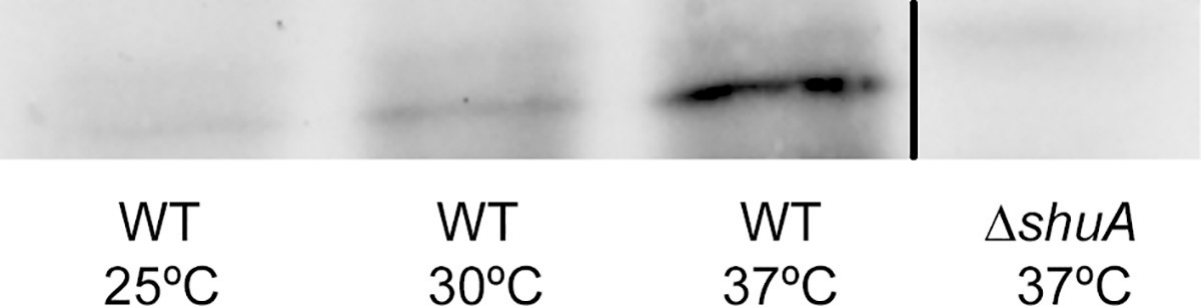
ShuA protein production is influenced by environmental temperature with increased levels detected at 37°C. Wild-type *S*. *dysenteriae* and *S*. *dysenteriae* Δ*shuA* knockout strain were grown to stationary phase at the temperatures indicated under iron limiting conditions (LB media containing 200 µg/mL EDDHA). Western blot analyses were conducted using whole-cell lysates generated from an equivalent number of bacteria grown under each condition and a polyclonal anti-*shuA* antibody (custom generated, Fisher Scientific). Results presented in this figure were spliced from the same underlying western blot experiment for which the underlying data are presented in the S1 File of this Correction notice.

**Fig 4:**
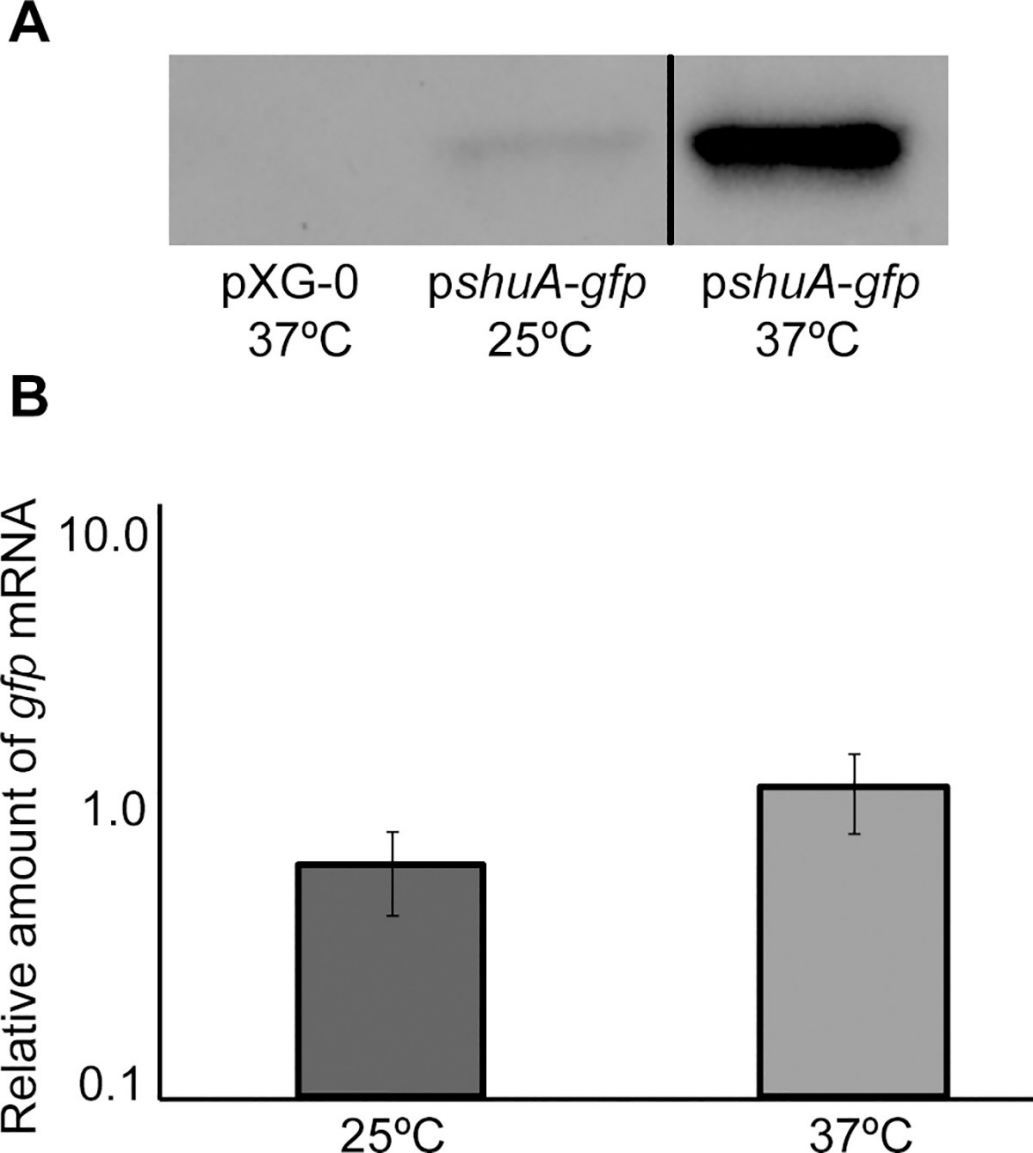
The *shuA* promoter and 5′ utr are sufficient to confer temperature-dependent post-transcriptional regulation. **A)** A Western blot analysis was performed with monoclonal anti-Gfp antibodies and whole-cell extracts generated from an equivalent number of wild-type *S*. *dysenteriae* carrying either p*shuA-gfp* or the empty vector pXG-0. All strains were cultured under iron-limited conditions (LB with 200 µg/mL EDDHA) to stationary phase at the temperatures indicated. Results shown in this panel were spliced from the same underlying western blot experiment for which the underlying data are presented in the S2 File of this Correction notice. **B)** Quantitative Real-time PCR was carried out using RNA isolated from wild-type *S*. *dysenteriae* carrying p*shuA-gfp* cultured at the indicated temperature under iron-limited conditions (LB with 200 µg/mL EDDHA). *gfp* mRNA levels were normalized to the amount of *rrsA* measured in each sample and expressed relative to the amount of *gfp* transcript in the first 25°C sample. All data are representative of three biological replicates and error bars represent one standard deviation. Assuming a confidence interval of 95% (p≤0.05), no significant difference exists between the relative levels of *gfp* transcript measured from p*shuA-gfp* following growth of the strain at 25°C or 37°C.

**Fig 6:**
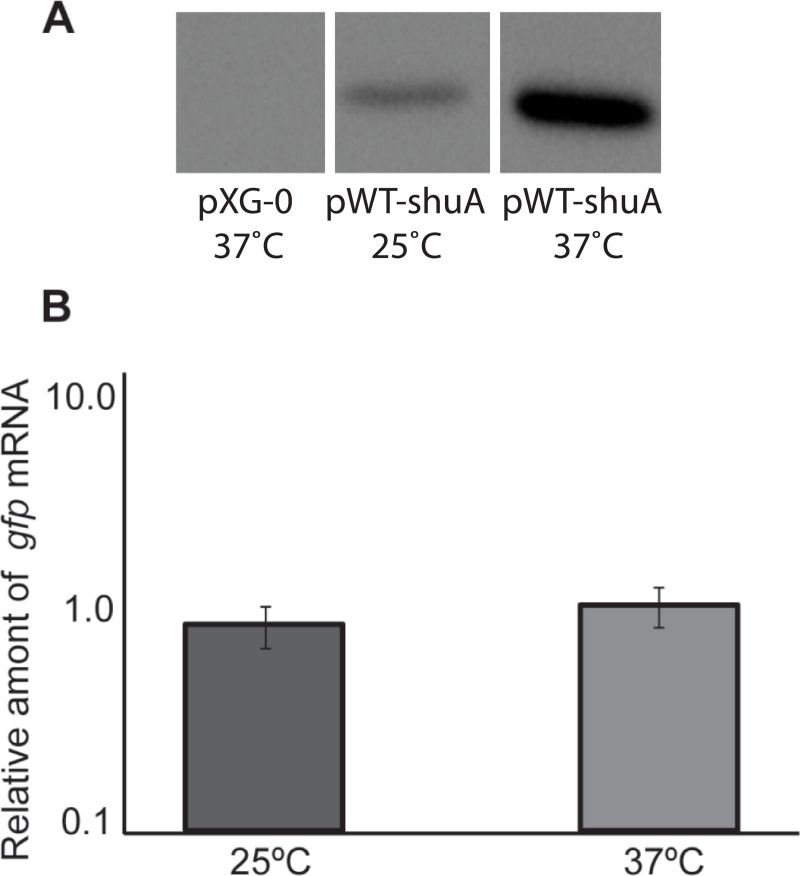
Sequences composing the *shuA* FourU thermometer are sufficient to confer post-transcriptional thermoregulation onto *gfp* expression. **A)** *E*. *coli* strain DH5α containing pWT-*shuA* or pXG-0, as a vector control, was cultured to stationary phase at the temperatures indicated. A Western blot was performed using whole-cell lysates generated from an equal number of cells and anti-Gfp monoclonal antibodies. Results shown in this panel were spliced from the same underlying western blot experiment for which the underlying data are presented in the S5 File of this Correction notice. **B)** Quantitative Real-time PCR was conducted using RNA isolated from *E*. *coli* DH5α carrying pWT-*shuA* following growth of the strain to stationary phase at 25°C or 37°C. *gfp* mRNA levels were normalized to *rrsA* measured in each sample and expressed relative to the amount of *gfp* transcript measured in the first 25°C sample. All data are representative of three biological replicates and error bars represent one standard deviation. Assuming a confidence interval of 95% (p≤0.05), no significant difference exists between the relative levels of *gfp* transcript measured from pWT-*shuA* following growth of the strain at 25°C or 37°C.

**Fig 7:**
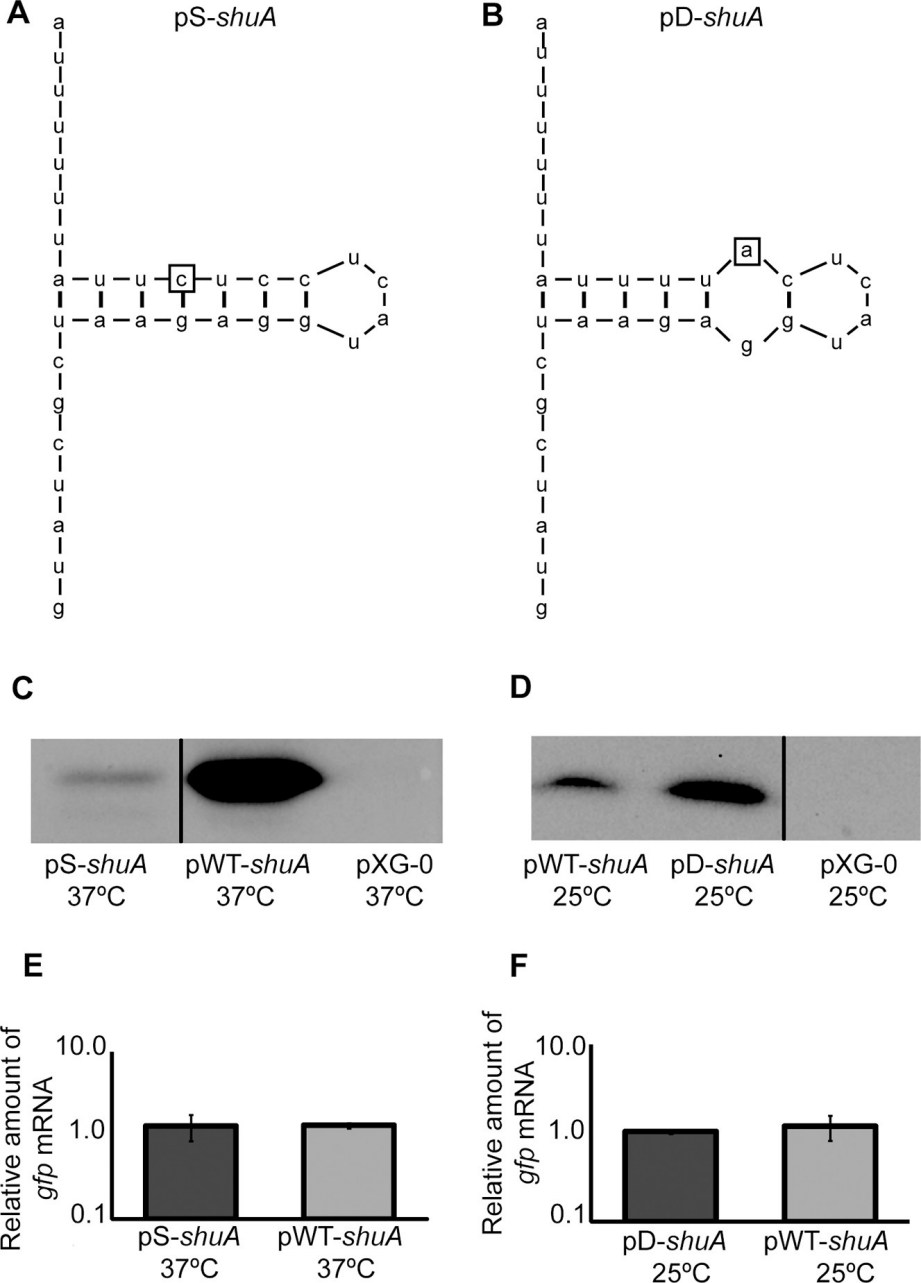
Mutational analysis demonstrates that the *shuA* 5′ utr contains a functional FourU RNA thermometer. The *shuA* region cloned into pWT-*shuA* was mutagenized to further validate the existence of a predicted FourU element within the *shuA* 5‘utr. **A)** The uracil residue located 19 nucleotides upstream of the *gfp* translational start site was mutated to a cytosine. This mutation, indicated by the box, is predicted to stabilize the inhibitory structure within the putative FourU RNA thermometer. This mutated sequence was cloned into the *gfp* translational reporter pXG-10 to generate the stabilized mutant construct designated pS-*shuA*. **B)** The cytosine residue 17 nucleotides upstream of the *gfp* translational start site was mutated to an adenine. This mutation, indicated by the box, is predicted to destabilize the inhibitory structure within the putative FourU RNA thermometer. This mutated sequence was cloned into the *gfp* translational reporter pXG-10 to generate the destabilized mutant construct designated pD-*shuA*. Western blot analyses were conducted using monoclonal anti-Gfp antibodies and whole-cell extracts generated from an equal number of *E*. *coli* carrying pWT-*shuA* or pS-*shuA* cultured to stationary phase in LB at the permissive temperature of 37°C **(C)**, and *E*. *coli* carrying pWT-*shuA* or pD-*shuA* cultured to stationary phase in LB at the non-permissive temperature of 25°C **(D).** Quantitative real-time PCR analysis was performed using RNA isolated from *E*. *coli* DH5α cells carrying pWT-*shuA*, pS-*shuA* and pD-*shuA* after culturing the strains to stationary phase using the temperatures indicated. *gfp* transcript levels were normalized to the amount of *rrsA* in each sample and set relative to the amount of *gfp* in the first pWT-*shuA* sample. All data are representative of three biological replicates and error bars represent one standard deviation. Assuming a confidence interval of 95% (p≤0.05), no significant difference exists between the relative levels of *gfp* transcript measured from pWT-*shuA* and pS-*shuA* **(E)** or pD-*shuA* **(F)** at the temperatures tested. Results shown in panels C and D were spliced from the same western blot experiments for which underlying data are presented in the S3 and S4 Files of this Correction notice respectively.

## Supporting information

S1 File Raw data underlying Fig 1. (TIF)

S2 File Raw data underlying Fig 4A. (TIF)

S3 File Raw data underlying Fig 7C. (TIF)

S4 File Raw data underlying Fig 7D. (TIF)

S5 File Raw data underlying corrected Fig 6A. (TIF)

S6 File Repeat experiments Fig 1. Western blot analyses using a custom generated anti-ShuA antibody and whole-cell lysates generated from equivalent numbers of wild-type *S*. *dysenteriae* or the *S*. *dysenteriae* Δ*shuA* knockout strain. All strains were cultured to the stationary phase of growth under iron limited conditions (LB media containing 200μg/ml EDDHA) at the indicated temperature. The arrow indicates the location of ShuA. The hand-drawn dashes in the first lane of the image indicate the location of the Precision Plus Protein Dual Color Standards (BioRad) present on the membrane (Not imaged upon expose of the blot to x-ray film). Data presented in this figure are in biological triplicate (Set 1, Set 2, and Set 3).
(PDF)

S7 File Repeat experiments Fig 4A. **A)** Western blot analyses using an anti-Gfp antibody and whole-cell lysates generated from an equivalent number of wild-type *S*. *dysenteriae* carrying either the p*shuA-gfp* reporter plasmid or the empty vector pXG-0. All strains were cultured to the stationary phase of growth under iron limited conditions (LB media containing 200μg/ml EDDHA) at the indicated temperature. **B)** An image of the membrane used in the above Western blot stained to show total protein content of each lane; included to demonstrate equivalent loading of each lane. Data presented in this figure are in biological duplicate (Set 1 and Set 2). Precision Plus Protein Dual Color Standard (BioRad) was used as the protein size marker in these assays (S). Note that the bright spots on each image result from a defect in the imaging systems and are not on the membranes themselves.
(PDF)

S8 File Repeat experiments Fig 6A. **A)** Western blot analyses using an anti-Gfp antibody and whole-cell lysates generated from an equivalent number of *E*. *coli* carrying either the pWT-*shuA* reporter plasmid or the empty vector pXG-0. All strains were cultured to the stationary phase of growth at the indicated temperature. **B)** An image of the membrane used in the above Western blot stained to show total protein content of each lane; included to demonstrate equivalent loading of each lane. Precision Plus Protein Dual Color Standard (BioRad) was used as the protein size marker in these assays (S). Data presented in this figure are in biological duplicate (Set 1 and Set 2). Note that the bright spots on each image result from a defect in the imaging systems and are not on the membranes themselves.
(PDF)

S9 File Repeat experiments Fig 7C. **A)** Western blot analyses using an anti-Gfp antibody and whole-cell lysates generated from an equivalent number of *E*. *coli* carrying pWT-*shuA*, pS-*shuA* or the empty vector pXG-0 following growth to stationary phase at the permissive temperature of 37˚C. **B)** An image of the membrane used in the above Western blot stained to show total protein content of each lane; included to demonstrate equivalent loading of each lane. Data presented in this figure are in biological duplicate (Set 1 and Set 2). Precision Plus Protein Dual Color Standard (BioRad) was used as the protein size marker in these assays (S). Note that the bright spots on each image result from a defect in the imaging systems and are not on the membranes themselves. S10 File. Repeat experiments Fig 7D.
(PDF)

S10 File Repeat experiments Fig 7D. **A)** Western blot analyses using an anti-Gfp antibody and whole-cell lysates generated from an equivalent number of *E*. *coli* carrying pWT-*shuA*, pD-*shuA* or the empty vector pXG-0 following growth to stationary phase at the non-permissive temperature of 25˚C. **B)** An image of the membrane used in the above Western blot stained to show total protein content of each lane; included to demonstrate equivalent loading of each lane. Data presented in this figure are in biological duplicate (Set 1 and Set 2). Precision Plus Protein Dual Color Standard (BioRad) was used as the protein size marker in these assays (S). Note that the bright spots on each image result from a defect in the imaging systems and are not on the membranes themselves.
(PDF)
