# Supplementary figures and images for: RNA-Mediated Thermoregulation of Iron-Acquisition Genes in Shigella dysenteriae and Pathogenic Escherichia coli
Source: PLoS One. 2013 May 21;8(5):e63781. doi: 10.1371/journal.pone.0063781 (PMC3660397; doi:10.1371/journal.pone.0063781)

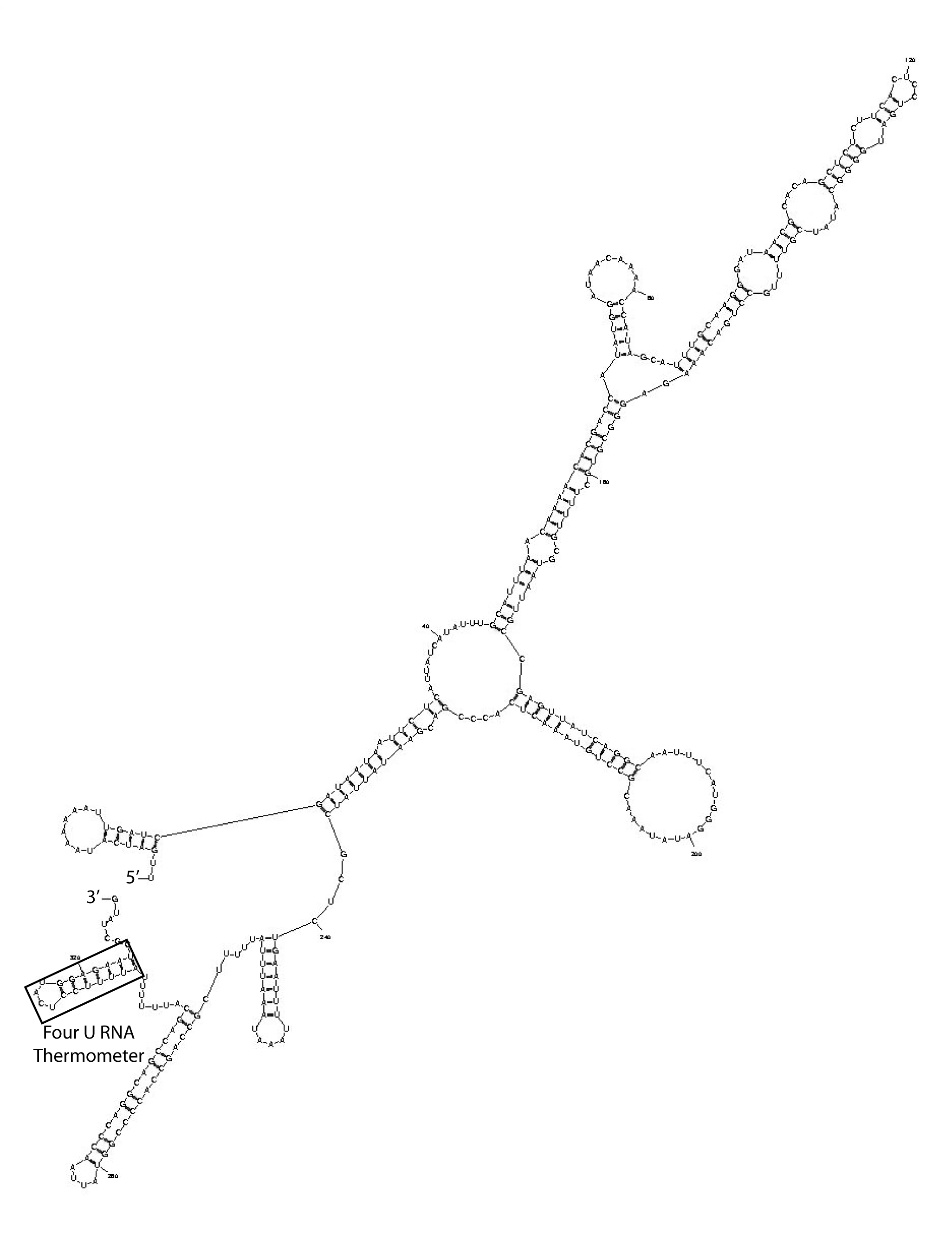

Supplement: Figure S1 — In-silico folding analysis of the full-length shuA 5′ utr. The full-length 5′ utr and start codon of shuA was submitted to Mfold for modeling of the RNA secondary structure. The predicted FourU RNA thermometer is denoted by a box. (TIF) [file pone.0063781.s001.tif]
