# Supplementary figures and images for: Correction draft: RNA-Mediated Thermoregulation of Iron-Acquisition Genes in Shigella dysenteriae and Pathogenic Escherichia coli
Source: PLoS One. 2021 Jun 1;16(6):e0252744. doi: 10.1371/journal.pone.0252744 (PMC8168886; doi:10.1371/journal.pone.0252744)

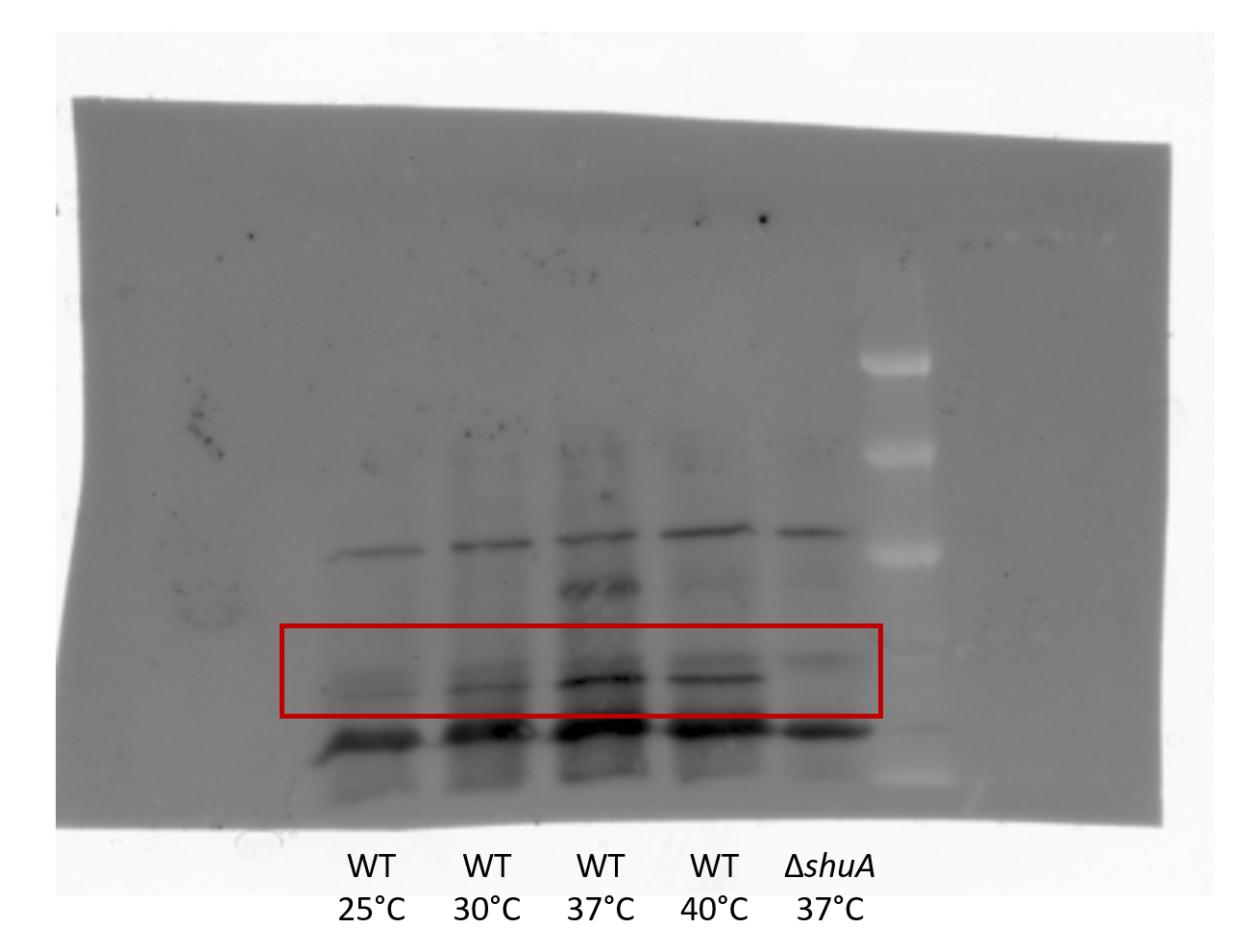

Supplement: S1 File — (TIF) [file pone.0252744.s001.tif]

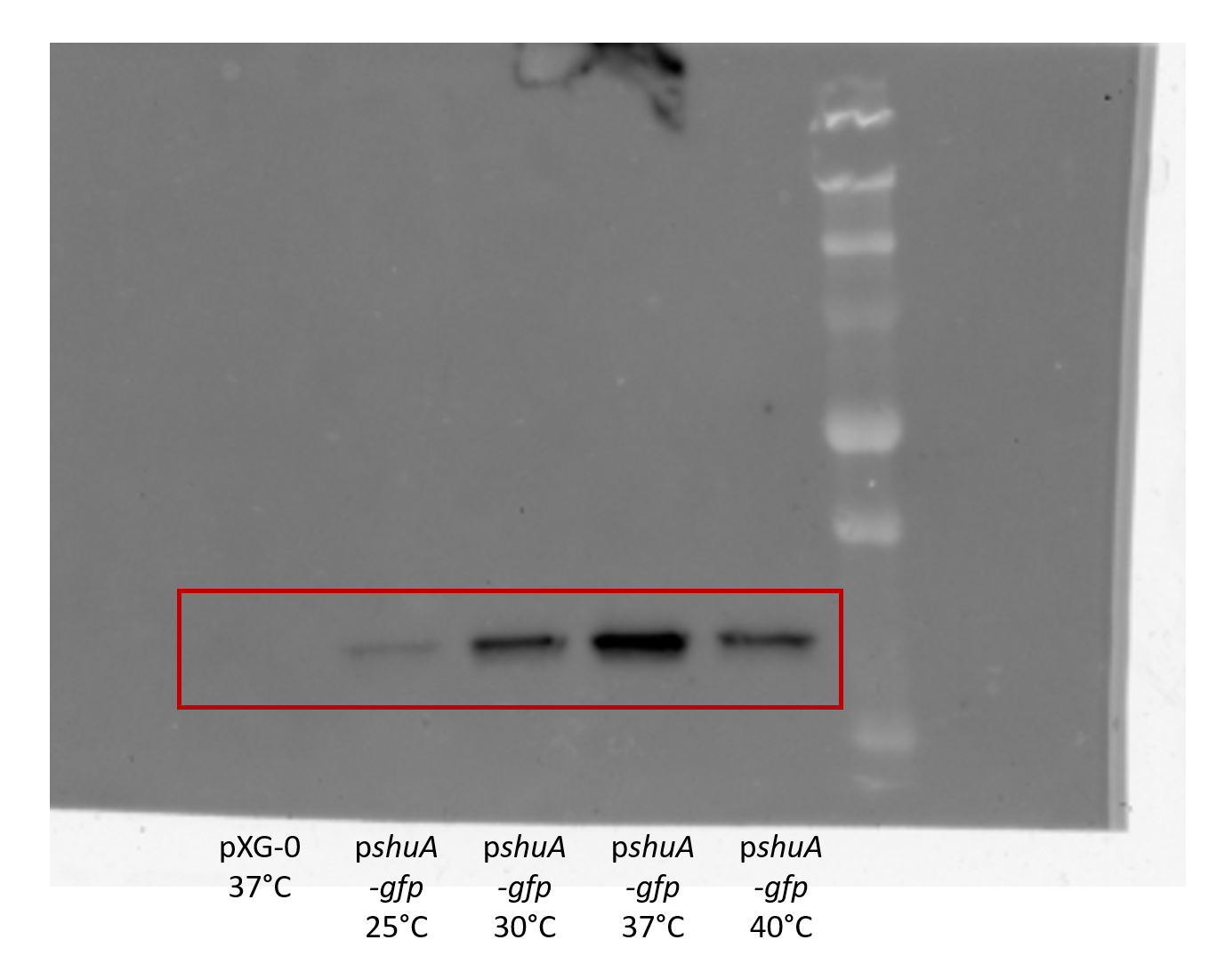

Supplement: S2 File — (TIF) [file pone.0252744.s002.tif]

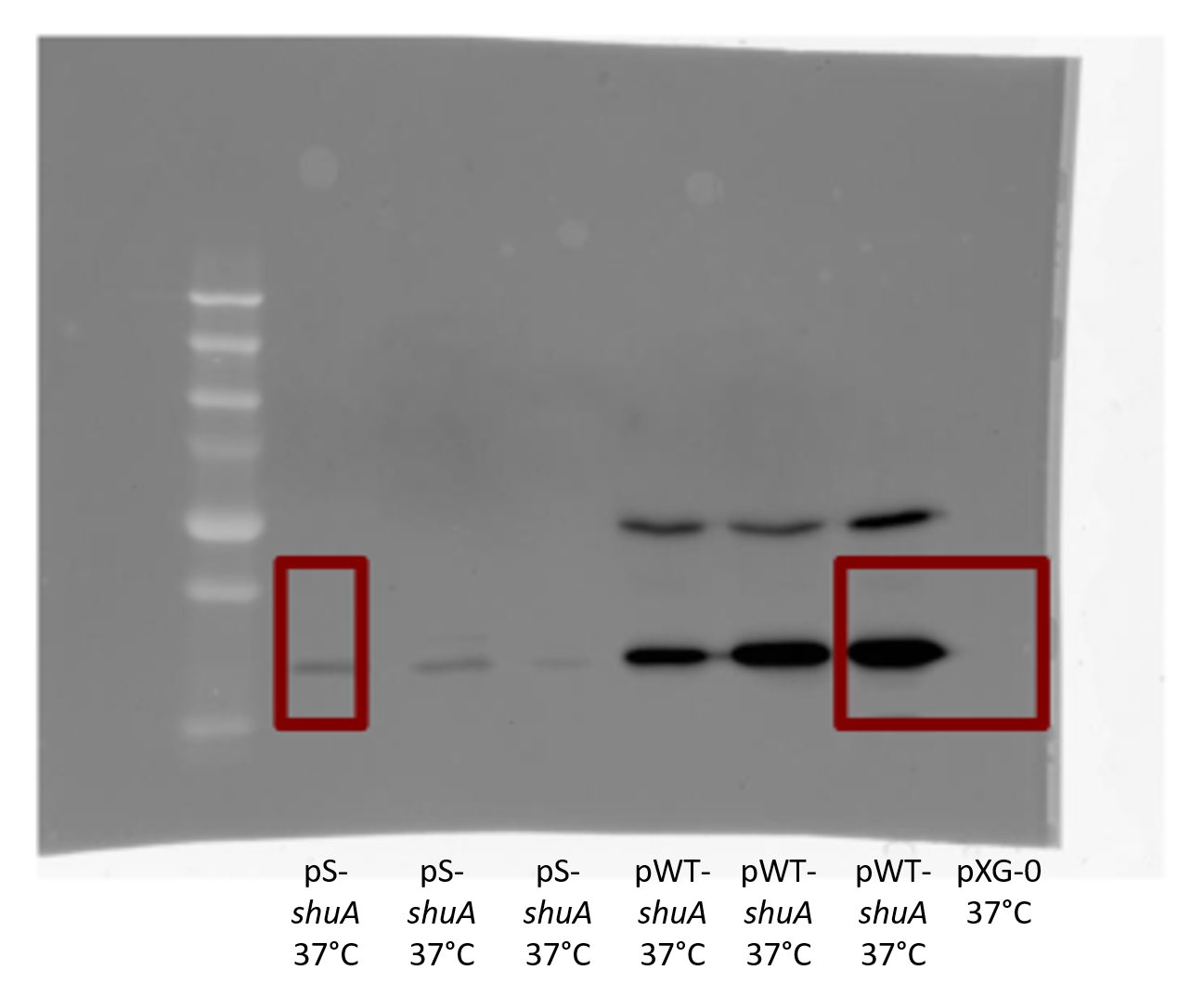

Supplement: S3 File — (TIF) [file pone.0252744.s003.tif]

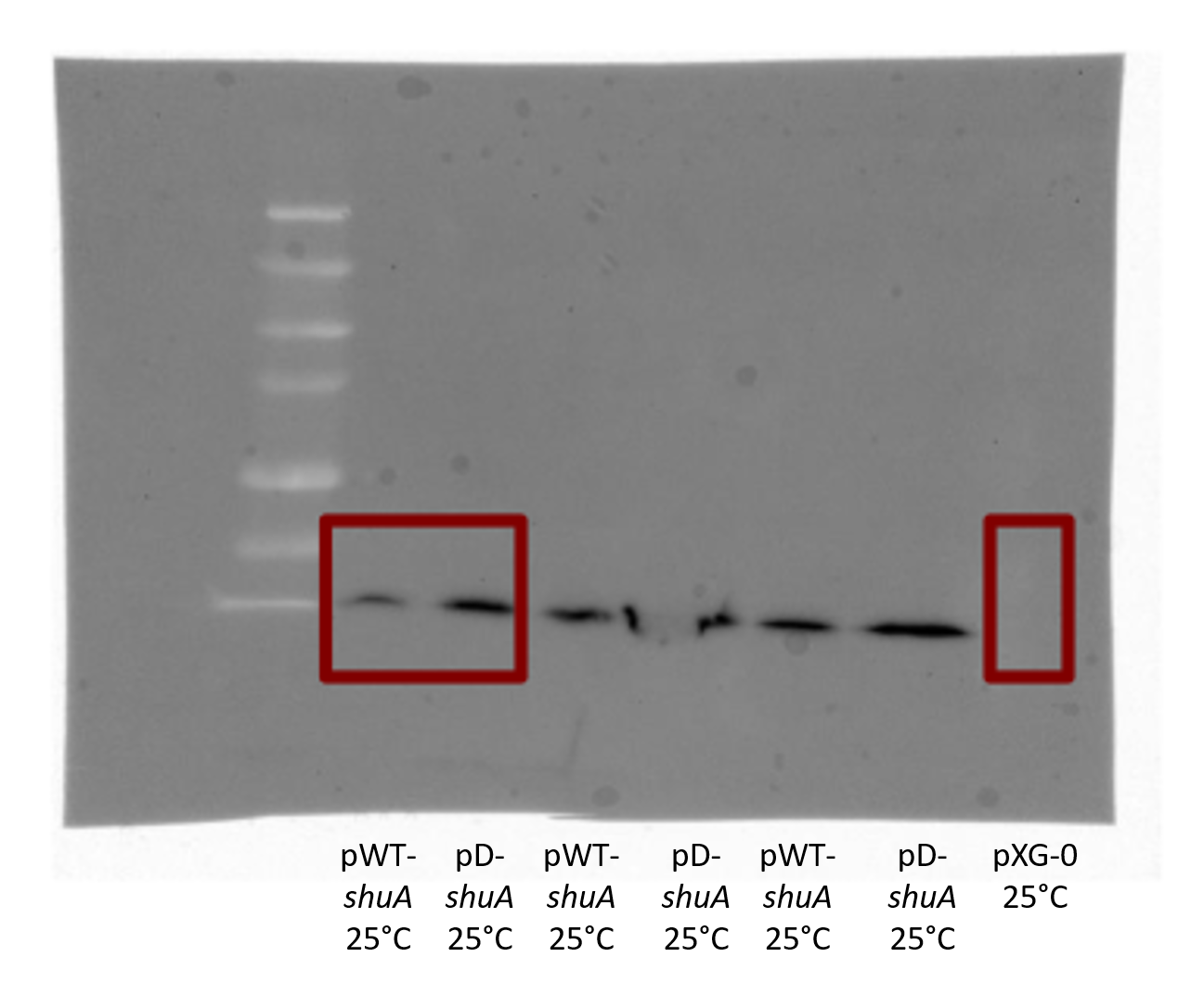

Supplement: S4 File — (TIF) [file pone.0252744.s004.tif]

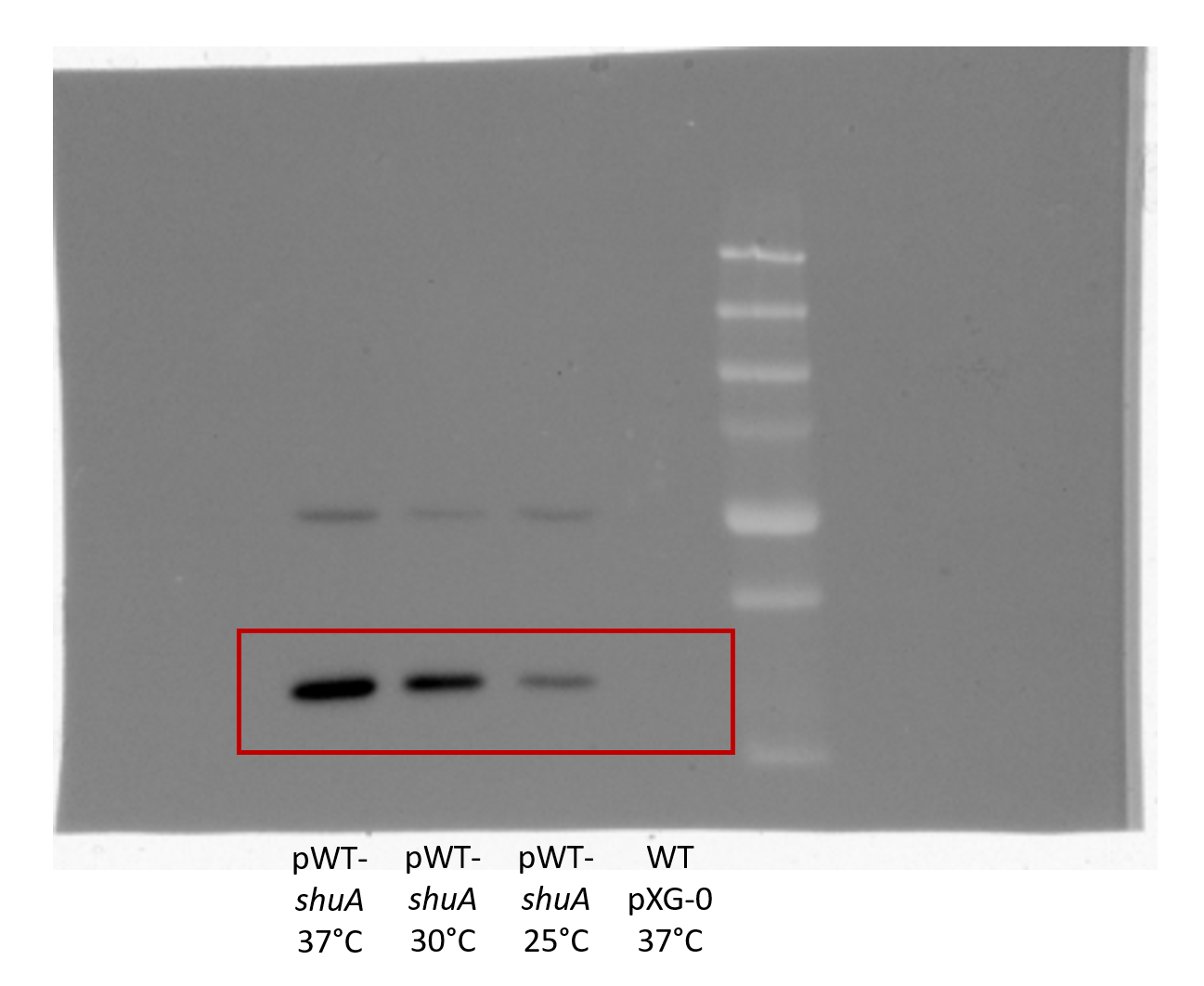

Supplement: S5 File — (TIF) [file pone.0252744.s005.tif]
