# Supplementary material for: Correction draft: RNA-Mediated Thermoregulation of Iron-Acquisition Genes in Shigella dysenteriae and Pathogenic Escherichia coli
Source: PLoS One. 2021 Jun 1;16(6):e0252744. doi: 10.1371/journal.pone.0252744 (PMC8168886; doi:10.1371/journal.pone.0252744)
Supplement: S6 File — Western blot analyses using a custom generated anti-ShuA antibody and whole-cell lysates generated from equivalent numbers of wild-type S. dysenteriae or the S. dysenteriae ΔshuA knockout strain. All strains were cultured to the stationary phase of growth under iron limited conditions (LB media containing 200μg/ml EDDHA) at the indicated temperature. The arrow indicates the location of ShuA. The hand-drawn dashes in the first lane of the image indicate the location of the Precision Plus Protein Dual Color Standards (BioRad) present on the membrane (Not imaged upon expose of the blot to x-ray film). Data presented in this figure are in biological triplicate (Set 1, Set 2, and Set 3). (PDF) [file pone.0252744.s006.pdf]

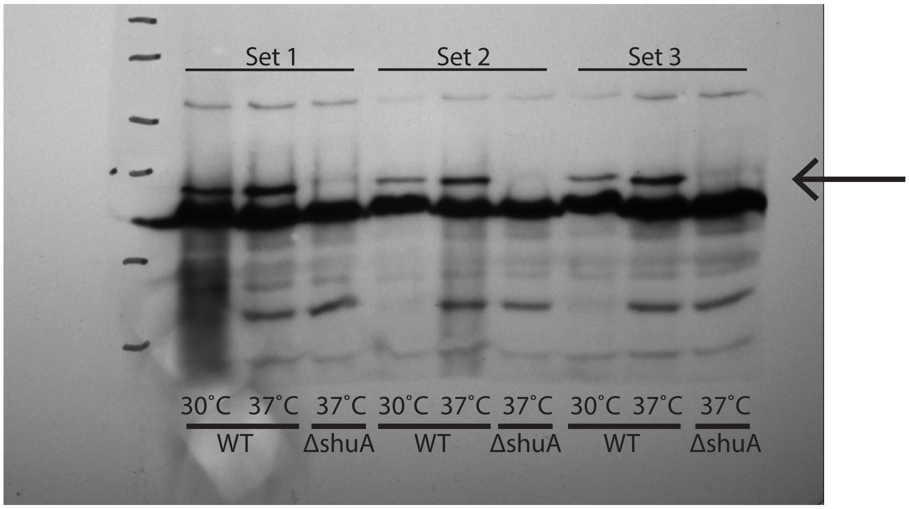

Additional Data in Support of Figure 1:

Western blot analyses using a custom generated anti-ShuA antibody and whole-cell lysates generated from equivalent numbers of wild-type *S. dysenteriae* or the *S. dysenteriae* Δ*shuA* knockout strain. All strains were cultured to the stationary phase of growth under iron limited conditions (LB media containing 200μg/ml EDDHA) at the indicated temperature. The arrow indicates the location of ShuA. The hand-drawn dashed in the first lane of the image indicate the location of the Precision Plus Protein Dual Color Standards (BioRad) present on the membrane (Not imaged upon expose of the blot to x-ray film). Data presented in this figure are in biological triplicate (Set 1, Set 2, and Set 3).
