# Supplementary material for: Correction draft: RNA-Mediated Thermoregulation of Iron-Acquisition Genes in Shigella dysenteriae and Pathogenic Escherichia coli
Source: PLoS One. 2021 Jun 1;16(6):e0252744. doi: 10.1371/journal.pone.0252744 (PMC8168886; doi:10.1371/journal.pone.0252744)
Supplement: S7 File — A) Western blot analyses using an anti-Gfp antibody and whole-cell lysates generated from an equivalent number of wild-type S. dysenteriae carrying either the pshuA-gfp reporter plasmid or the empty vector pXG-0. All strains were cultured to the stationary phase of growth under iron limited conditions (LB media containing 200μg/ml EDDHA) at the indicated temperature. B) An image of the membrane used in the above Western blot stained to show total protein content of each lane; included to demonstrate equivalent loading of each lane. Data presented in this figure are in biological duplicate (Set 1 and Set 2). Precision Plus Protein Dual Color Standard (BioRad) was used as the protein size marker in these assays (S). Note that the bright spots on each image result from a defect in the imaging systems and are not on the membranes themselves. (PDF) [file pone.0252744.s007.pdf]

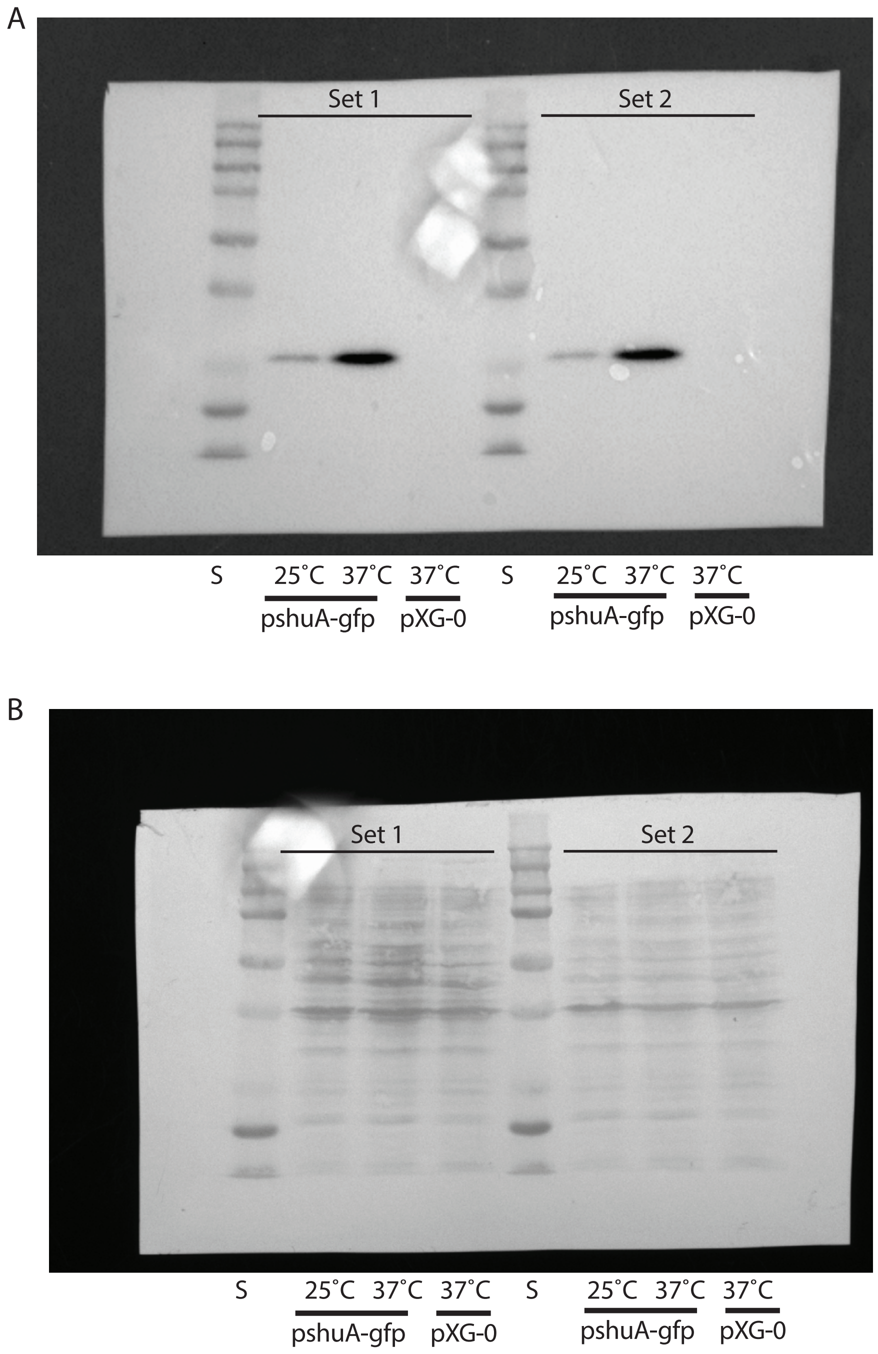


**Additional Data in Support of Figure 4A:**

**A)** Western blot analyses using an anti-Gfp antibody and whole-cell lysates generated from an equivalent number of wild-type *S. dysenteriae* carrying either the p*shuA-gfp* reporter plasmid or the empty vector pXG-0. All strains were cultured to the stationary phase of growth under iron limited conditions (LB media containing 200µg/ml EDDHA) at the indicated temperature. **B)** An image of the membrane used in the above Western blot stained to show total protein content of each lane; included to demonstrate equivalent loading of each lane. Data presented in this figure are in biological duplicate (Set 1 and Set 2). Precision Plus Protein Dual Color Standard (BioRad) was used as the protein size marker in these assays (S). Note that the bright spots on each image result from a defect in the imaging systems and are not on the membranes themselves.
