# Supplementary material for: Correction draft: RNA-Mediated Thermoregulation of Iron-Acquisition Genes in Shigella dysenteriae and Pathogenic Escherichia coli
Source: PLoS One. 2021 Jun 1;16(6):e0252744. doi: 10.1371/journal.pone.0252744 (PMC8168886; doi:10.1371/journal.pone.0252744)
Supplement: S8 File — A) Western blot analyses using an anti-Gfp antibody and whole-cell lysates generated from an equivalent number of E. coli carrying either the pWT-shuA reporter plasmid or the empty vector pXG-0. All strains were cultured to the stationary phase of growth at the indicated temperature. B) An image of the membrane used in the above Western blot stained to show total protein content of each lane; included to demonstrate equivalent loading of each lane. Precision Plus Protein Dual Color Standard (BioRad) was used as the protein size marker in these assays (S). Data presented in this figure are in biological duplicate (Set 1 and Set 2). Note that the bright spots on each image result from a defect in the imaging systems and are not on the membranes themselves. (PDF) [file pone.0252744.s008.pdf]

A

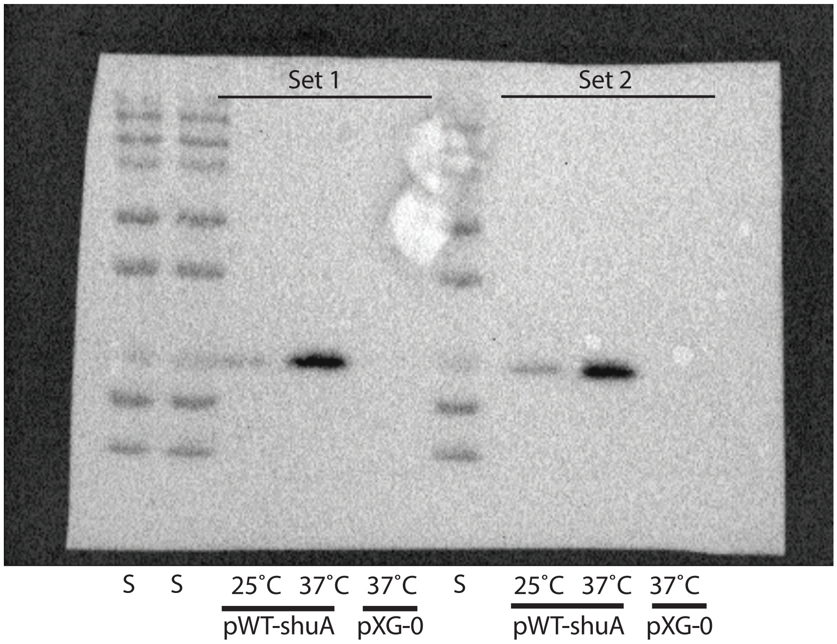

B

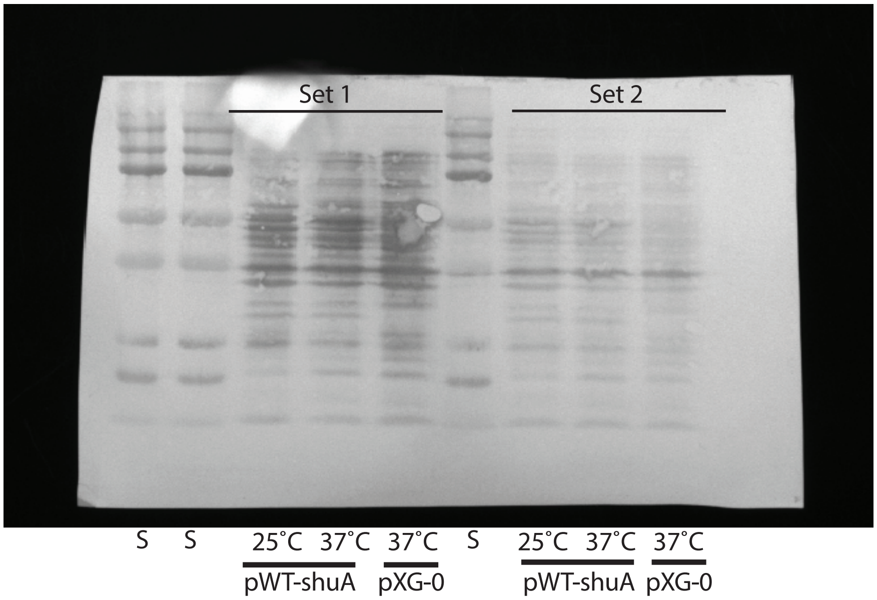

Additional Data in Support of Figure 6A:

A) Western blot analyses using an anti-Gfp antibody and whole-cell lysates generated from an equivalent number of *E. coli* carrying either the pWT-*shuA* reporter plasmid or the empty vector pXG-0. All strains were cultured to the stationary phase of growth at the indicated temperature. **B)** An image of the membrane used in the above Western blot stained to show total protein content of each lane; included to demonstrate equivalent loading of each lane. Precision Plus Protein Dual Color Standard (BioRad) was used as the protein size marker in these assays (S). Data presented in this figure are in biological duplicate (Set 1 and Set 2). Note that the bright spots on each image result from a defect in the imaging systems and are not on the membranes themselves.
