# Supplementary material for: Correction draft: RNA-Mediated Thermoregulation of Iron-Acquisition Genes in Shigella dysenteriae and Pathogenic Escherichia coli
Source: PLoS One. 2021 Jun 1;16(6):e0252744. doi: 10.1371/journal.pone.0252744 (PMC8168886; doi:10.1371/journal.pone.0252744)
Supplement: S10 File — A) Western blot analyses using an anti-Gfp antibody and whole-cell lysates generated from an equivalent number of E. coli carrying pWT-shuA, pD-shuA or the empty vector pXG-0 following growth to stationary phase at the non-permissive temperature of 25˚C. B) An image of the membrane used in the above Western blot stained to show total protein content of each lane; included to demonstrate equivalent loading of each lane. Data presented in this figure are in biological duplicate (Set 1 and Set 2). Precision Plus Protein Dual Color Standard (BioRad) was used as the protein size marker in these assays (S). Note that the bright spots on each image result from a defect in the imaging systems and are not on the membranes themselves. (PDF) [file pone.0252744.s010.pdf]

A

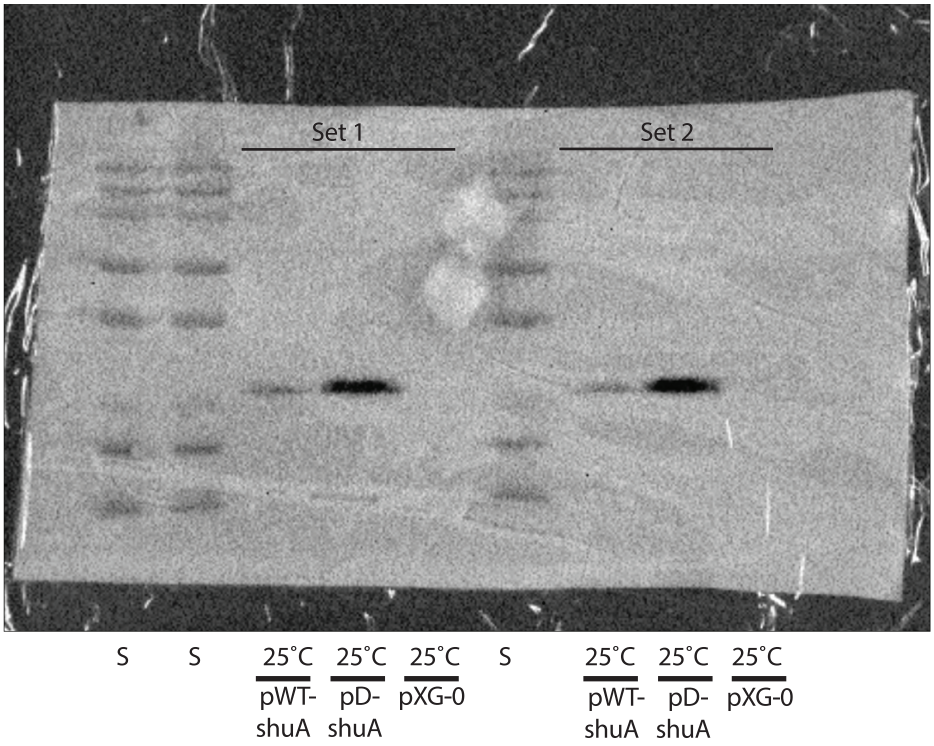

B

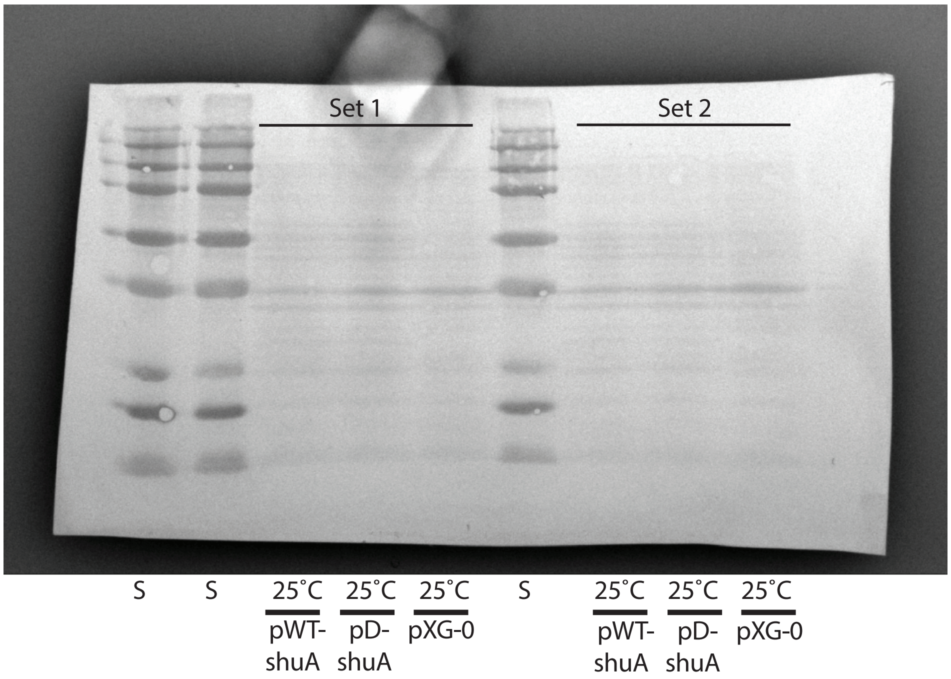

Additional Data in Support of Figure 7D:

A) Western blot analyses using an anti-Gfp antibody and whole-cell lysates generated from an equivalent number of *E. coli* carrying pWT-*shuA*, pD-*shuA* or the empty vector pXG-0 following growth to stationary phase at the non-permissive temperature of 25˚C. **B)** An image of the membrane used in the above Western blot stained to show total protein content of each lane; included to demonstrate equivalent loading of each lane. Data presented in this figure are in biological duplicate (Set 1 and Set 2). Precision Plus Protein Dual Color Standard (BioRad) was used as the protein size marker in these assays (S). Note that the bright spots on each image result from a defect in the imaging systems and are not on the membranes themselves.
